# Enhancing the Comprehensive Performance and Interfacial Adhesion of Emulsified Asphalt Using an Epoxy-Functionalized Waterborne Polyurethane

**DOI:** 10.3390/polym18060719

**Published:** 2026-03-16

**Authors:** Yifan Liu, Zhenhao Cao, Minghao Mu, Zheng Wang, Jia Wang, Yanyan Zhang, Kunyu Wang, Yang Liu, Xue Li

**Affiliations:** 1 School of Chemistry and Chemical Engineering, University of Jinan, 336 West Road of Nan Xinzhuang, Jinan 250022, China; lyfujn@163.com (Y.L.); chm_caozh@163.com (Z.C.); wj13593042686@163.com (J.W.); zyy0010301@163.com (Y.Z.);; 2 Shandong Hi-Speed Group Innovation Research Institute, No. 8, Long’ao North Road, Lixia District, Jinan 250100, China; 3 Shandong Academy of Environmental Sciences Co., Ltd., 1277 Zhenyuan Street, Jinan 250100, China; 4 Jinan Daoerdao New Material Technology Co., Ltd., 408-7 East Unit, Jinan University Science and Technology Park, 988 Shunxing Road, Jinan 250031, China

**Keywords:** waterborne polyurethane, modified emulsified asphalt, interfacial adhesion, chemical modification, road performance

## Abstract

To enhance the comprehensive performance and interfacial adhesion of conventional emulsified asphalt, an epoxy-functionalized waterborne polyurethane modified emulsified asphalt (EFPU-MEA) was developed using an epoxy-functionalized waterborne polyurethane (EFPU) emulsion and an isocyanate curing agent. Experimental evaluations show that the EFPU-MEA achieves a tensile strength of 1.11 ± 0.05 MPa and an elongation at break of 782.5 ± 45%, demonstrating a well-balanced flexibility and deformation resistance. The interfacial bond between EFPU-MEA and aggregates exhibited robust durability under various stressors, including thermal fluctuations, low-temperature cracking, chemical corrosion, and moisture damage. Quantitative “sandwich” pull-out and shear tests determined the optimal modifier content and spraying quantity to be 15–20% and 1.0 kg/m^2^, respectively. Under these conditions, the system maintained high bond strength following severe freeze–thaw cycles and chemical erosion. Mechanistically, fluorescence microscopy (FM) confirmed a uniform dispersion of EFPU within the asphalt matrix, providing effective physical reinforcement. Furthermore, surface free energy (SFE) analysis and Fourier Transform Infrared (FTIR) spectroscopy revealed that internal chemical crosslinking restructures the binder’s surface thermodynamics, significantly increasing the surface polarity and adhesion work. Finally, road performance tests—including marshall stability, wet track abrasion, and rutting resistance—verified the engineering durability of the EFPU-MEA mixture. These findings provide a theoretical and practical basis for the use of EFPU-MEA in extending the service life of high-grade highway pavements.

## 1. Introduction

The long-term service performance of asphalt pavements is predominantly determined by the inherent properties of the asphalt binder and the adhesion strength at the asphalt–aggregate interface [1]. In recent years, cold-mix emulsified asphalt technology has gained increasing attention in road construction and maintenance due to its energy efficiency and environmental benefits. However, conventional emulsified asphalt often exhibits poor durability against weathering. Under complex environmental conditions, such as moisture infiltration and temperature fluctuations, the interfacial adhesion between the asphalt and aggregate deteriorates significantly [2], severely limiting its engineering application. This limitation primarily stems from the dependence of conventional emulsified asphalt on weak interfacial interactions, such as physical adsorption and van der Waals forces, to maintain bonding. These interactions are vulnerable to environmental stressors, leading to early-stage distresses like stripping and raveling [3,4]. Consequently, this vulnerability represents a critical bottleneck hindering the application of emulsified asphalt in high-grade highways.

To mitigate these challenges, polymer modification has emerged as a prevalent solution for upgrading emulsified asphalt. Current literature is dominated by physical blending approaches utilizing conventional latexes. Notable studies by He et al. [5], Fan et al. [6], and Li et al. [7] demonstrated that blending styrene–butadiene rubber (SBR) latex with waterborne epoxy resin (WER) yields a synergistic effect, combining the elasticity of SBR with the adhesiveness of WER to enhance service performance. Furthermore, Wang et al. [8], Fu et al. [9], and Zhou et al. [10] utilized SBS latex to create a network within the binder, significantly improving the thermal stability and elastic recovery. Nevertheless, although physical blending enhances the binder’s bulk properties, it fails to address the fundamental limitation of low adhesion strength. This deficiency stems from the non-polar nature of polymers such as SBS and SBR, which interact with asphalt and aggregates primarily through weak physical mechanisms like van der Waals forces [11]. As a result, the interface is vulnerable to stripping under environmental stressors such as moisture damage and temperature fluctuations. This vulnerability poses an intrinsic limitation to the efficacy of conventional physical blending strategies.

Waterborne polyurethane (WPU) has emerged as a compelling modifier due to its exceptional mechanical properties, eco-compatibility, and highly tunable molecular structure. Distinct from non-polar polymers, WPU chains are rich in polar functional groups, such as hydroxyl, carbamate (urethane), and potentially residual isocyanate groups [12]. These active groups offer a solid theoretical basis for establishing strong interfacial adhesion and forming chemical bridges between the polyurethane chains and the asphalt matrix. Nevertheless, the prevailing research paradigm remains largely confined to physical blending strategies. For example, Wang et al. [13] incorporated a WPU/waterborne epoxy resin (WER) blend into micro-surfacing, reporting marked enhancements in mechanical and adhesive properties. Zhou et al. [14] further revealed, through molecular dynamics and experimental analysis, that WPU improves adhesion primarily by amplifying physical van der Waals forces. Additionally, Fan et al. [15] engineered a high-performance fog-seal material via the ternary blending of WPU, WER, and SBR. However, while these studies demonstrate the utility of WPU, they fail to fully exploit its capacity for chemical interaction. The absence of robust chemical crosslinking between WPU and the asphalt binder restricts the efficiency of the modification, leaving the material’s full potential unrealized.

Traditionally, the adhesion of emulsified asphalt is assessed using qualitative protocols like boiling or ultrasonic stripping tests [16,17]. However, these visual-based methods fail to provide precise data on bond strength. In contrast, quantitative approaches such as pull-out tests have been employed to measure the adhesion force [18,19]. Nevertheless, existing studies often conduct these tests on cement concrete slabs [20] rather than actual pavement aggregates. Such simplified substrates fail to capture the complex surface texture and mineralogy of real aggregates or the degradation of interfacial bonding in complex environments (e.g., freeze–thaw cycles and chemical erosion), thereby compromising the practical relevance of the evaluation results.

While previous studies have extensively explored the physical intermolecular blending of waterborne polyurethane and epoxy emulsions to modify asphalt, such conventional blended systems frequently encounter micro-phase separation and asynchronous curing issues during the complex demulsification and water evaporation stages. To overcome these fundamental thermodynamic bottlenecks, this study proposes a novel modification strategy utilizing epoxy-functionalized waterborne polyurethane (EFPU) [21] combined with a reactive isocyanate curing agent. Distinct from traditional physical blends, the EFPU system achieves a graft copolymer architecture where active epoxy groups are chemically grafted directly onto the polyurethane chain segments, effectively synergizing the flexibility of polyurethane with the high mechanical strength and exceptional adhesion of epoxy resin. Building on this foundation, the study further leverages the specific reactivity of isocyanate groups to chemically react with both the terminal hydroxyl groups of the EFPU chains and the active hydrogen simultaneously regarding chemical modification in functional groups within the asphalt binder [22], thereby acting as a “chemical bridge”. This internal chemical crosslinking fundamentally restructures the surface thermodynamics of the binder, triggering a massive surge in surface polarity to form an exceptionally robust polar adhesion network with the aggregate. To rigorously quantify this chemically enhanced cross-scale adhesion, a “sandwich” pull-out and shear test method using actual limestone aggregates was employed [23]. Unlike traditional qualitative assessments or tests on idealized substrates, this method enables a realistic and quantitative evaluation of bond strength evolution under simulated harsh environmental conditions.

On this basis, this study systematically investigates the basic properties of EFPU-modified emulsified asphalt (EFPU-MEA) and evaluates its interfacial adhesion performance through quantitative analysis combined with interfacial failure observation [24]. Subsequently, the underlying modification mechanisms are elucidated via fluorescence microscopy, surface free energy (SFE) analysis, and Fourier Transform Infrared (FTIR) spectroscopy. Finally, the road performance is verified to ensure its engineering applicability. The findings are expected to provide new insights into optimizing the comprehensive performance and adhesion of emulsified asphalt and offer a theoretical basis for addressing interfacial failures in high-grade highway applications.

## 2. Materials and Methods

### 2.1. Raw Materials

The base emulsified asphalt utilized in this study was prepared in the laboratory using 70^#^ paving asphalt (supplied by Sinopec Qilu Company Ltd., Zibo, Shandong, China). Its fundamental properties were characterized in strict accordance with the Standard Test Methods of Bitumen and Bituminous Mixtures for Highway Engineering (JTG E20-2011) [25].

To elucidate the chemical basis for the modification mechanism, the chemical composition of the base asphalt was further analyzed. As summarized in Table 1, the binder exhibits a robust SARA composition characteristic of high-quality paving asphalt, with a high content of aromatics (52.40%) ensuring good compatibility with modifiers. Crucially, the total content of polar fractions (resins: 20.60%; asphaltenes: 19.80%) reaches 40.40%. Together with a measured acid value of 0.764 mg KOH/g, this confirms the presence of active hydrogen functional groups (e.g., -OH, -NH-, and -COOH) within the asphalt matrix. As established in the recent literature, these highly polar fractions, particularly asphaltenes, inherently contain reactive hydrogen groups such as carboxylic acids, amines, and phenols. While their overall concentration is relatively low compared to the non-polar hydrocarbon bulk, they serve as essential reactive sites for chemical crosslinking with the isocyanate curing agent to form carbamate linkages [26].

All technical indices met the relevant specification requirements. The epoxy-functionalized waterborne polyurethane (EFPU) emulsion was synthesized in-house via a tailored acetone method. While inspired by the foundational framework previously reported by our research group [21], the specific synthetic route and formulations were entirely redesigned for this study. For comparison, the polyurethane curing agent was sourced from Shanghai Sisheng Polymer Materials Co., Ltd. (Shanghai, China), while the styrene–butadiene rubber (SBR) latex (used as a control) was commercially obtained from Shandong Square Sea Rubber Technology Co., Ltd. (Zibo, Shandong, China). Additionally, styrene–butadiene–styrene (SBS) latex, serving as another control modifier, was prepared in the laboratory using commercial SBS 4303 polymer (styrene/butadiene mass ratio of 30/70) according to a previously established method [27]. The relevant physicochemical properties of these materials are detailed in Table 2, Table 3 and Table 4, respectively. The limestone aggregates employed in the experiments were also obtained from a provider in Shandong Province.

### 2.2. Synthesis of Hydroxyl-Terminated EFPU Emulsion

**Figure 1:**
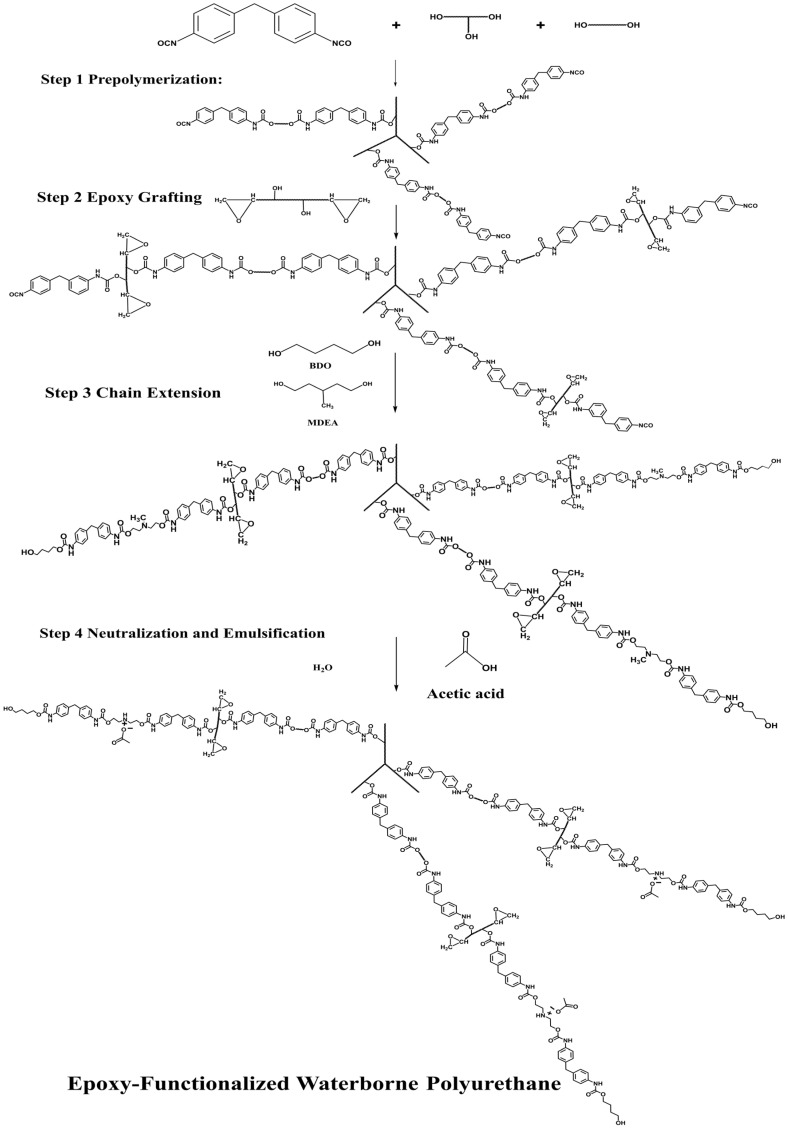
Synthetic route of EFPU.

The synthesis of the cationic hydroxyl-terminated EFPU emulsion was conducted via a stepwise “acetone process,” as illustrated in Figure 1. The specific formulation is detailed in Table 5.

Step 1 Pre-polymerization: Polytetramethylene ether glycol (PTMG, Mn = 1000) and Castor Oil (C.O) were dehydrated at 110 °C under vacuum for 1.5 h to remove trace moisture. After cooling the mixture to 50 °C, the temperature of MDI-50 (a commercial mixture comprising approximately 50% 4,4′-diphenylmethane diisocyanate and 50% 2,4′-diphenylmethane diisocyanate with an NCO content of approximately 33.6 wt%, supplied by Wanhua Chemical Group Co., Ltd., Yantai, China) was then raised to 60 °C, and the reaction was maintained for 2 h. At this stage, the NCO content was monitored via di-n-butylamine titration to ensure the pre-polymerization reached the theoretical NCO value (NCO excess) before proceeding. At this stage, the reaction progress was monitored via standard di-n-butylamine titration. Pre-polymerization was deemed complete when the actual measured NCO content reached 12.85 wt%, which closely approximated our theoretical target value of 13.00 wt%. This confirmed the successful synthesis of the isocyanate-terminated pre-polymer before proceeding to the subsequent grafting stage.

Step 2 Epoxy Grafting: Bisphenol-A-type epoxy resin E-20 (solid at room temperature, with an experimentally determined epoxy value of 0.204 ± 0.002 mol/100 g via standard hydrochloric acid–acetone titration) was first dissolved in anhydrous acetone to prepare a 50 wt% solution. This solution was introduced into the NCO-terminated pre-polymer, and the mixture was stirred at 45 °C for 1 h.

Step 3 Chain Extension: The hydrophilic chain extender N-methyl diethanolamine (MDEA) and small molecule extender 1,4-Butanediol (BDO) were dissolved in a minimal amount of anhydrous acetone and added to the grafted pre-polymer. The reaction continued at 45 °C for 2 h. These extenders consumed the remaining NCO groups, and the overall stoichiometry of the entire formulation was controlled to achieve a final NCO/OH ratio of 0.95, ensuring the final synthesized polymer chains were completely terminated with hydroxyl groups.

Step 4 Neutralization and Emulsification: The system was cooled to 30 °C. Acetic Acid (HAc) was added to neutralize the tertiary amine groups of MDEA (110% neutralization degree) over 30 min. Subsequently, deionized water was added under high-speed shearing (2000 rpm) to induce phase inversion, transforming the water-in-oil dispersion into an oil-in-water emulsion. Finally, acetone was removed via rotary evaporation to yield the EFPU emulsion.

### 2.3. Preparation of Modified Emulsified Asphalt (MEA) Samples

**Figure 2:**
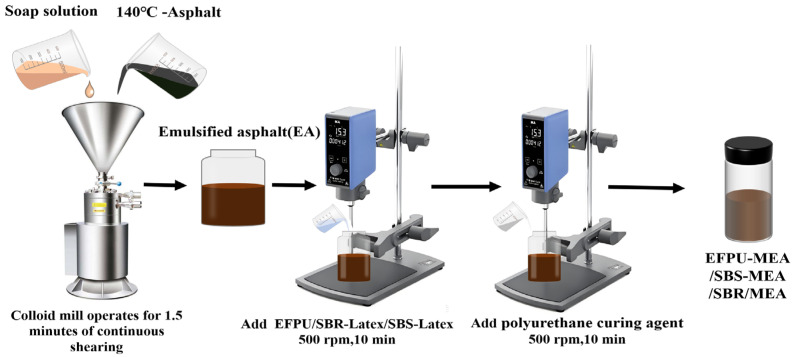
Process flow diagram of modified emulsified asphalt preparation.

Under a stirring speed of 500 rpm, a predetermined amount of EFPU was added at a constant rate into the emulsified asphalt (EA) and mixed continuously for 10 min. Subsequently, a polyurethane curing agent equivalent to 5% of the EFPU mass was introduced. This specific dosage was optimized based on a sensitivity analysis of the pure polymer films (see Figure S1 in Supplementary Materials), which revealed that 5% achieves the optimal equilibrium between tensile strength and flexibility. While higher dosages continued to increase strength, they caused a drastic reduction in elongation at break, rendering the material too brittle for flexible pavement applications. Following this addition, the mixture was stirred for an additional 10 min to obtain the EFPU-modified emulsified asphalt (EFPU-MEA). By adjusting the EFPU content (5%, 10%, 15%, 20%, and 25%), a series of samples were prepared and labeled accordingly as EFPU-MEA-5, EFPU-MEA-10, EFPU-MEA-15, EFPU-MEA-20, and EFPU-MEA-25. The preparation process is illustrated in Figure 2. For comparative purposes, control samples were also prepared, including EA modified with 4% SBS emulsion (designated as SBS-MEA-4) and EA modified with 3% SBR emulsion (designated as SBR-MEA-3).

### 2.4. Methods

#### 2.4.1. Basic Performance

A series of performance characteristics, including mechanical properties, heat resistance, low-temperature flexibility, resistance to chemical media corrosion, and adhesion to aggregate, were systematically evaluated for the prepared EFPU-MEA samples. The tests were conducted according to the following standards: mechanical properties (GB/T 528-2009 [28]), heat resistance and low-temperature performance (JC/T 975-2005 [29]), resistance to acid and alkali corrosion (GB/T 16777-2008 [30]), and adhesion (JTG E20-2011).

(1) Mechanical Properties of EFPU-MEA

**Figure 3:**
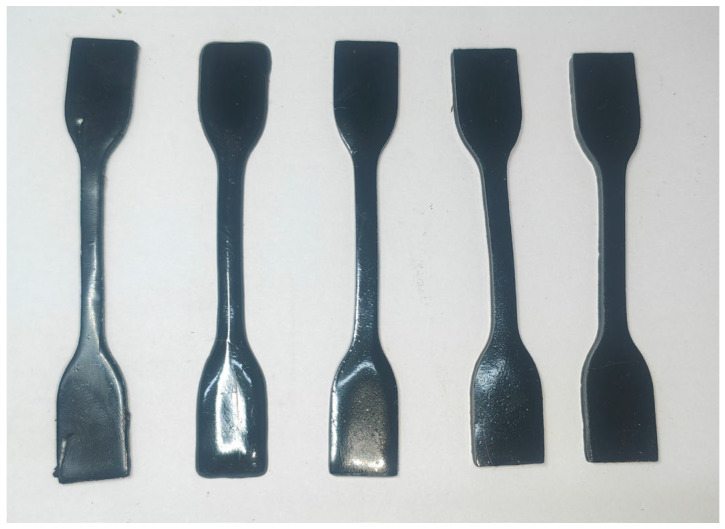
Mechanical property test specimen.

The mechanical properties of the EFPU-MEA, including tensile strength (TS) and elongation at break (E_b_), were tested via a direct tensile method. Firstly, free-standing films of the emulsion residue were prepared following a low-temperature film-forming method for emulsified asphalt [31]. Subsequently, these films were cut into dumbbell-shaped specimens of specified dimensions using a standard cutter (as shown in Figure 3). The tensile strength and elongation at break were calculated according to Equations (1) and (2), respectively:

(1) $$TS=\frac{F_{m}}{wt}$$

(2) $$E_{b}=\frac{100\left(L_{b}-L_{0}\right)}{L_{0}}$$

where F_m_ is the maximum force recorded (N); w is the thickness at the narrow section of the specimen (mm); t is the width at the narrow section of the cutter (mm); L_0_ is the original gauge length (mm); and L_b_ is the gauge length at break (mm).

(2) Heat Resistance of EFPU-MEA

**Figure 4:**
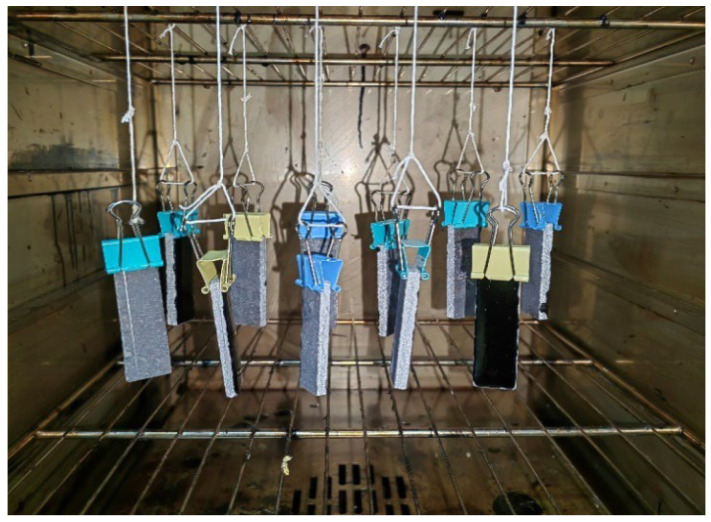
Heat resistance test.

The heat resistance was evaluated by uniformly applying the EFPU-MEA samples with different modifier contents, along with control groups, onto limestone substrates (100 mm × 20 mm) at a spraying quantity of 1.0 kg/m^2^. After complete curing and drying, the initial mass (m_0_) of the specimens was recorded. The specimens were then vertically suspended in an oven and maintained at 80 °C, 100 °C, and 120 °C for 5 h, respectively. Following this thermal treatment, the coating surface was examined for any flow or dripping phenomena. The final mass (m_1_) was subsequently measured. The mass retention rate (R_H_) was calculated using Equation (3). The setup for the heat resistance test is illustrated in Figure 4:

(3) $$R_{H}=\frac{m_{1}}{m_{0}}100\%$$

where R_H_ is the mass retention rate (%); m_0_ is the initial mass of the specimen (g); and m_1_ is the final mass of the specimen (g).

(3) Low-Temperature Flexibility of EFPU-MEA

**Figure 5:**
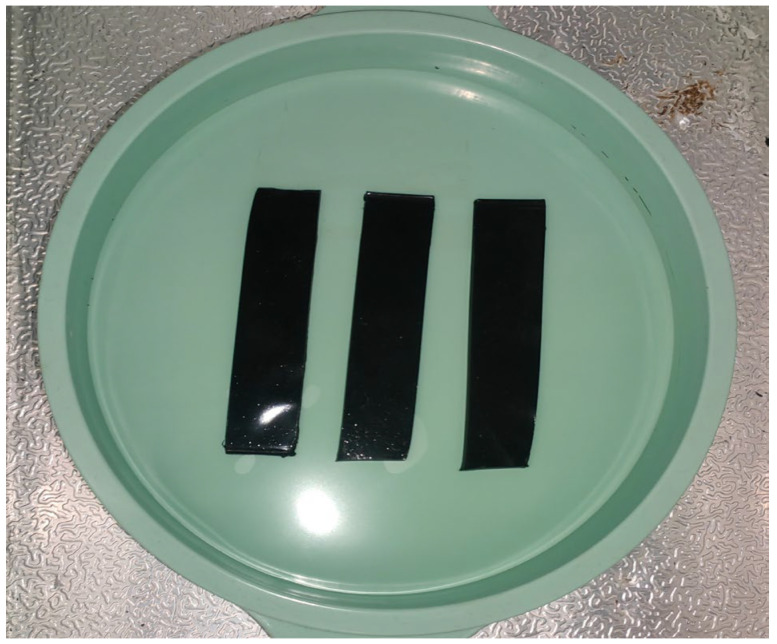
Freeze–thaw resistance test.

The low-temperature flexibility was evaluated by cutting the prepared EFPU-MEA and control group films into standard specimens measuring 100 mm × 25 mm × (1.5 ± 0.2) mm. These specimens, along with a 15 mm diameter steel rod, were conditioned at −15 °C for 2 h (as shown in Figure 5). Subsequently, each specimen was uniformly bent 360°around the rod within 3 s and examined for surface cracking or brittle fracture. Each test was performed in triplicate.

(4) Chemical Media Corrosion Resistance of EFPU-MEA

**Figure 6:**
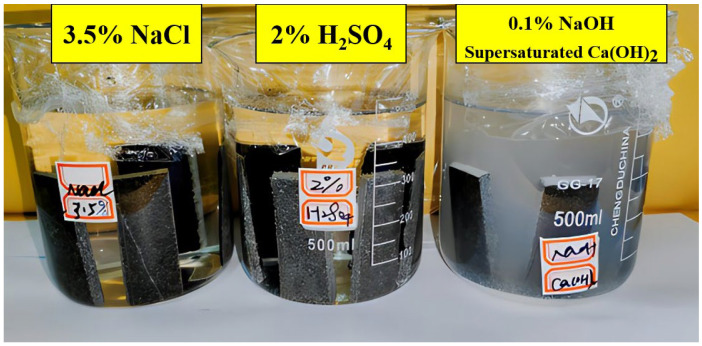
Corrosion resistance in acid, alkali, and salt tests.

The resistance to chemical media corrosion was evaluated by uniformly applying the prepared EFPU-MEA samples and control groups onto limestone substrates (100 mm × 20 mm). After complete curing, the specimens were immersed in different corrosive media. The media included a 2% sulfuric acid solution (acidic), an alkaline solution prepared with 0.1% NaOH and a saturated Ca(OH)_2_ solution, and a 3.5% NaCl solution. Triplicate sets of parallel specimens were individually immersed in 400 mL of the corresponding solution for 72 h, with the liquid level maintained at least 10 mm above the top of the specimens. Upon completion of the immersion, the specimens were removed, gently rinsed with deionized water, and dried at room temperature. The coatings were then examined for signs of deterioration, such as peeling, blistering, discoloration, tackiness, or efflorescence. The experimental setup for the chemical corrosion resistance test is depicted in Figure 6.

(5) Conventional Adhesion Performance of EFPU-MEA

The adhesion performance between different samples and coarse aggregates was evaluated using the boiling water test. The specific procedure was as follows: Clean, dry coarse aggregates with a particle size range of 13.2–19 mm was first immersed in deionized water for 1 min, followed by complete submersion in the respective emulsified asphalt samples for another 1 min. After removal, the coated aggregates were cured under ventilated conditions for 24 h. Subsequently, the samples were immersed in boiling water and boiled for 3 min. The adhesion performance was assessed by visually inspecting and rating the state of the asphalt film stripping. The water bath temperature was strictly controlled throughout the test to ensure uniform heating of the asphalt coating. Each test was conducted with two parallel replicates.

#### 2.4.2. Pull-Out and Shear Performance Tests

Limestone, being the primary aggregate utilized in the subsequent macroscopic asphalt mixtures, was deliberately employed as the testing substrate. Unlike standard bond tests that typically use metallic stubs, this specific material matching allows for a direct and authentic quantitative evaluation of the true chemical interactions, polar adhesion, and micro-mechanical interlocking between the EFPU-MEA binder and the actual aggregate surface.

**Figure 7:**
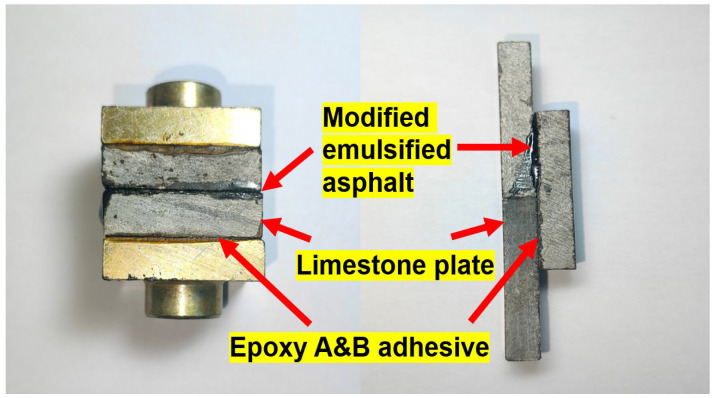
Schematic diagram of the pull-out and shear test specimens.

The specimen preparation procedure was as follows: Limestone was cut into 40 mm × 40 mm × 10 mm blocks, and the bonding surfaces were ground with 200-grit sandpaper, followed by cleaning and drying. A uniform layer of EFPU-MEA was then applied between the bonding surfaces of two prepared blocks. Loading plates were symmetrically bonded to the outer surfaces, resulting in a “limestone–asphalt–limestone” sandwich-type composite structure (Figure 7). After preparation, the sandwich specimens were directly placed into a 35 °C environmental chamber and conditioned until reaching a constant weight. This specific temperature (35 °C) was deliberately selected to realistically simulate the typical pavement surface temperatures during summer construction seasons, ensuring the complete evaporation of water, full demulsification, and the natural formation of the crosslinked asphalt film under field-equivalent conditions.

To investigate the influence of environmental factors, after determining the optimal spraying quantity and EFPU content, the tests were conducted under various service conditions, including high/low temperatures, water immersion, and freeze–thaw cycles. For each specific test condition, five parallel specimens (*n* = 5) were prepared and tested to ensure data reproducibility and statistical reliability. Both pull-out and shear tests were performed using a universal testing machine at a loading rate of 100 mm/min to evaluate the true interlayer pull-out and shear resistance under simulated traffic loading, inspired by established methodologies in the recent literature [32]. The pull-out strength and shear strength were calculated according to Equations (4) and (5), respectively:

(4) $$\sigma =\frac{P_{max}}{A}$$

(5) $$\tau =\frac{F_{max}}{M}$$

where σ is the pull-out strength (MPa); P_max_ is the maximum pull-out force (kN); A is the effective pull-out area (m^2^); τ is the shear strength (MPa); F_max_ is the maximum shear force (kN); and M is the effective shear area (m^2^).

(1) Determination of the optimal spraying quantity

To investigate the influence of the asphalt spraying quantity on the bond performance (pull-out and shear strength) at the asphalt–aggregate interface and to identify the spraying quantity for subsequent experiments, the EFPU-MEA-20 formulation, which exhibited superior overall performance, was selected for this investigation. This selection was made to isolate the variable of interest and exclude potential interference from insufficient modifier content. Specimens were prepared at spraying quantities of 0.4, 0.6, 0.8, 1.0, 1.2, and 1.6 kg/m^2^. The effects of these rates on the pull-out strength and shear strength were systematically evaluated.

(2) Effect of EFPU content

The EFPU content directly influences the chemical activity and cohesive properties of the modified asphalt. Insufficient content would limit the modification effectiveness and the number of active groups, thereby weakening the interfacial bonding. Conversely, excessively high content could lead to the inhomogeneous dispersion of EFPU within the asphalt, resulting in performance degradation. Therefore, a gradient of EFPU contents—namely, EFPU-MEA-5, EFPU-MEA-10, EFPU-MEA-15, EFPU-MEA-20, and EFPU-MEA-25—was designed, and the corresponding specimens were prepared for systematic testing.

(3) Effect of ambient temperature

To evaluate the influence of temperature on the bond performance, the specimens were conditioned at 10 °C, 5 °C, 25 °C, 45 °C, and 65 °C in an environmental chamber for 2 h to investigate the variations in pull-out and shear strengths under different temperature conditions.

(4) Effect of water immersion environment

To investigate the degradation of interfacial bond performance due to moisture erosion, specimens were fully immersed in a water bath maintained at 25 °C, with the water level kept at least 50 mm above the specimens. A magnetic stirrer was used to simulate water flow scour. Samples were taken and subjected to pull-out and shear tests after 24 h and 48 h of immersion, respectively.

(5) Effect of freeze–thaw cycles

To evaluate the impact of freeze–thaw damage on the bond performance, the specimens were first saturated by immersing them in water at 25 °C for 24 h. Subsequently, they were conditioned at −10 °C for 16 h (freezing phase), followed by thawing at 25 °C for 12 h (thawing phase), constituting one complete freeze–thaw cycle. The pull-out performance was tested immediately following the completion of the freeze–thaw cycles.

#### 2.4.3. Fourier Transform Infrared (FTIR) Spectrum

Attenuated Total Reflectance–Fourier Transform Infrared Spectroscopy (ATR-FTIR, Bruker Inventio S) was employed to characterize the chemical functional group composition of the EFPU-MEA samples. For testing, a small amount of the sample was uniformly applied to the diamond crystal stage. Spectra were acquired over the wavenumber range of 500–4000 cm^−1^ with a resolution of 2 cm^−1^, and 64 scans were accumulated to enhance the signal-to-noise ratio. Analysis of the assignment and shifts in the characteristic absorption peaks was conducted to reveal the chemical changes occurring in EFPU during the modification process.

#### 2.4.4. Fluorescent Microscope (FM) Observation

Fluorescence microscopy (Leica DM2500, Wetzlar, Germany) was employed to characterize the distribution state of EFPU within the asphalt phase by examining the microscopic phase morphology of EFPU-MEA with different modifier contents. The sample preparation procedure was as follows: EFPU-MEA was uniformly coated onto glass slides. After complete curing, the dispersion morphology and distribution homogeneity of EFPU within the emulsified asphalt were directly observed under the fluorescence microscope.

#### 2.4.5. Contact Angle Test

The surface free energy of different samples was determined using contact angle measurements, from which the work of adhesion, work of debonding, and energy ratio were calculated. The contact angles of formamide, glycerol, and distilled water on both the modified emulsified asphalt samples and the limestone substrate were measured at 25 °C using the pendant drop method with a contact angle goniometer [33]. Each measurement was performed in triplicate, and the average values were used for subsequent calculations.

Based on surface energy theory [34,35], the surface tension of solids and liquids comprises dispersive and polar components, which can be calculated using Equations (6) and (7), respectively. The solid–liquid interfacial free energy is given by Equation (8), while Young’s equation (Equation (9)) describes the relationship between the surface free energy and contact angle. Following the established Owens–Wendt framework, the surface energy calculation formula based on dispersive and polar components (Equation (10)) was applied. As demonstrated in other studies [32], this methodological approach is highly effective for correlating surface polarity with adhesion performance in modified bitumen binders:

(6) $$\gamma_{l}=\gamma_{l}^{d}+\gamma_{l}^{p}$$

(7) $$\gamma_{s}=\gamma_{s}^{d}+\gamma_{s}^{P}$$

(8) $$\gamma_{sl}=\gamma_{s}+\gamma_{l}-2\sqrt{\gamma_{s}^{d}\gamma_{s}^{d}}-2\sqrt{\gamma_{s}^{p}\gamma_{l}^{p}}$$

(9) $$\gamma_{l}\cos\theta =\gamma_{s}+\gamma_{sl}$$

(10) $$\frac{\left(1+\cos\theta \right)}{2}\frac{\gamma_{l}}{\sqrt{\gamma_{l}^{d}}}=\sqrt{\gamma_{s}^{p}}\sqrt{\frac{\gamma_{l}^{P}}{\gamma_{l}^{d}}}+\sqrt{\gamma_{s}^{d}}$$

where $\gamma_{l}$ is the surface free energy of liquid (mJ/m^2^); $\gamma_{s}$ is the surface free energy of solids (mJ/m^2^); $\gamma_{sl}$ is the surface free energy of the solid–liquid interface (mJ/m^2^); $\gamma_{l}^{d}$ is the polar component of the surface energy of liquids (mJ/m^2^); $\gamma_{s}^{d}$ is the dispersion component of solid surface energy; and $\gamma_{s}^{p}$ is the polar component of solid surface energy.

The cohesion of asphalt was evaluated using the cohesive energy (Equation (11)), with higher values indicating greater resistance to deformation and cracking. The asphalt–aggregate interfacial bonding performance was assessed by the work of adhesion (Equation (12)), representing the bond strength in dry conditions, and the work of debonding (Equation (13)), characterizing the stability under water immersion conditions. The energy ratio (Equation (14)) was employed to evaluate water resistance, where larger values suggest superior anti-stripping performance:

(11) $$w_{c}=2\gamma_{a}$$

(12) $$w_{as}=\gamma_{a}+\gamma_{S}-\gamma_{as}=2\sqrt{\gamma_{a}^{d}\gamma_{s}^{d}}+2$$

(13) $$w_{aws}=w_{aw}-w_{as}+w_{sw}=2\left(\sqrt{\gamma_{a}^{d}\gamma_{w}^{d}}+\sqrt{\gamma_{a}^{p}\gamma_{w}^{p}}+\sqrt{\gamma_{s}^{d}\gamma_{w}^{d}}+\sqrt{\gamma_{s}^{p}\gamma_{w}^{p}}-\sqrt{\gamma_{a}^{d}\gamma_{s}^{d}}-\sqrt{\gamma_{a}^{p}\gamma_{S}^{p}}\right)$$

(14) $$ER=\left|\frac{w_{as}}{w_{aws}}\right|$$

where $w_{c}$ is the cohesive energy of asphalt (mJ/m^2^); $\gamma_{a}$ is the surface free energy of asphalt (mJ/m^2^); $\gamma_{as}$ is the surface free energy of the asphalt–aggregate system (mJ/m^2^); $w_{as}$ is the adhesion work at the asphalt–aggregate interface (mJ/m^2^); $\gamma_{a}^{d}$ is the dispersive component of asphalt surface energy (mJ/m^2^); $\gamma_{a}^{p}$ is the polar component of asphalt surface energy (mJ/m^2^); $\gamma_{s}^{d}$ is the dispersive component of the surface energy of aggregates (mJ/m^2^); $\gamma_{s}^{p}$ is the polar component of aggregate surface energy (mJ/m^2^); $w_{aws}$ is the work of adhesion at the asphalt–aggregate interface (mJ/m^2^); $\gamma_{w}^{d}$ is the dispersion component of the surface energy of water (mJ/m^2^); and $\gamma_{w}^{p}$ is the polar component of the surface energy of water (mJ/m^2^).

Based on the calculated work of adhesion, the interfacial bond strength between EFPU-MEA and limestone aggregate under dry conditions can be evaluated; the work of debonding characterizes the interfacial stability under moisture erosion, while the energy ratio thereby provides a comprehensive assessment of the system’s resistance to water damage.

#### 2.4.6. Marshall Stability Test

**Figure 8:**
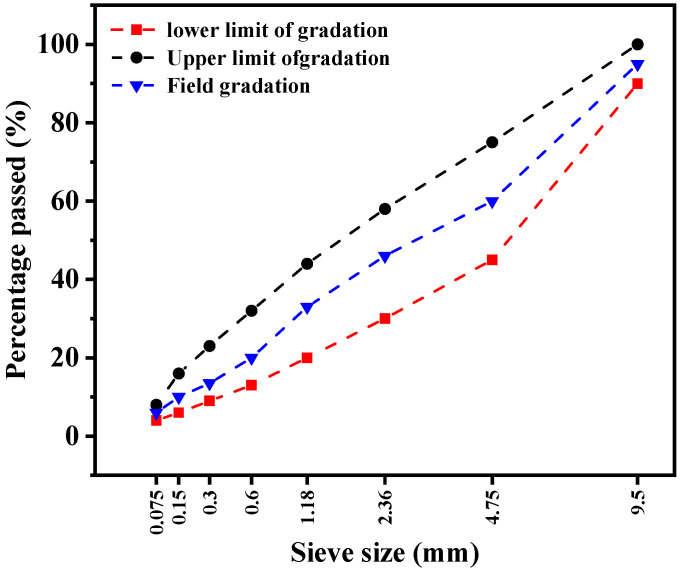
Gradation curve of the synthetic gradation mixture.

Cold-mix asphalt mixtures were prepared using aggregates blended to a custom synthetic gradation designed to resemble a micro-surfacing structure. The specific gradation curve is illustrated in Figure 8. The water damage resistance and adhesion performance of the EFPU-modified mixtures were evaluated via Marshall stability tests using a Marshall stability tester, in accordance with the standard DB11/T 2257-2024 [36].

#### 2.4.7. Wet Track Abrasion Test (WTAT)

To evaluate the interfacial adhesion and water damage resistance of the EFPU-modified emulsified asphalt (EFPU-MEA), wet track abrasion test (WTAT) specimens were prepared using the aforementioned custom synthetic gradation. The testing procedure was conducted in strict accordance with the standard method (T 0752) specified in JTG E20-2011, and the abrasion loss was calculated using Equation (15):

(15) $$WTAT=\left(m_{a}-m_{b}\right)∕A$$

where WTAT is the abrasion loss value of the specimen (g/m^2^); m_a_ is the mass of the specimen before abrasion (g); m_b_ is the mass of the specimen after abrasion (g); and A is the abrasion area of the rubber hose in the abrasion head, taken as 0.0304 m^2^.

#### 2.4.8. Anti-Rutting Performance

The high-temperature rutting resistance of the EFPU-MEA mixture was characterized using a wheel tracking test. The mixture was prepared using the aforementioned custom synthetic gradation and placed into a rutting slab mold measuring 300 mm × 300 mm × 50 mm. It was first allowed to stand in a ventilated environment at room temperature for 30 min to facilitate initial demulsification. Subsequently, the mixture was compacted using a wheel rolling compactor and then cured in a forced-air oven at 60 °C for 72 h to ensure complete moisture evaporation and strength development. After curing, the specimens were cooled and stored at room temperature. Prior to testing, the slabs were conditioned in a constant temperature chamber at 60 °C for at least 4 h. The wheel contact pressure was set to 0.7 ± 0.05 MPa. Three replicate specimens were prepared for each group, and the average dynamic stability (DS) was calculated.

#### 2.4.9. Statistical Analysis

Quantitative experiments, specifically the mechanical property, pull-out, and shear tests, were performed with five parallel replicates (*n* = 5) to ensure high reproducibility. The experimental results are expressed as the mean value ± standard deviation (SD). The statistical significance of the differences between groups was evaluated using a one-way analysis of variance (ANOVA) followed by Tukey’s post hoc test. A value of *p* < 0.05 was considered statistically significant. In the presented figures, different letters indicate statistically significant differences among the sample groups. In the presented figures, different lowercase letters (e.g., a, b, c) and uppercase letters (e.g., A, B, C) are used to indicate statistically significant differences among the sample groups under different testing conditions or evaluation factor.

## 3. Results and Discussions

**Figure 9:**
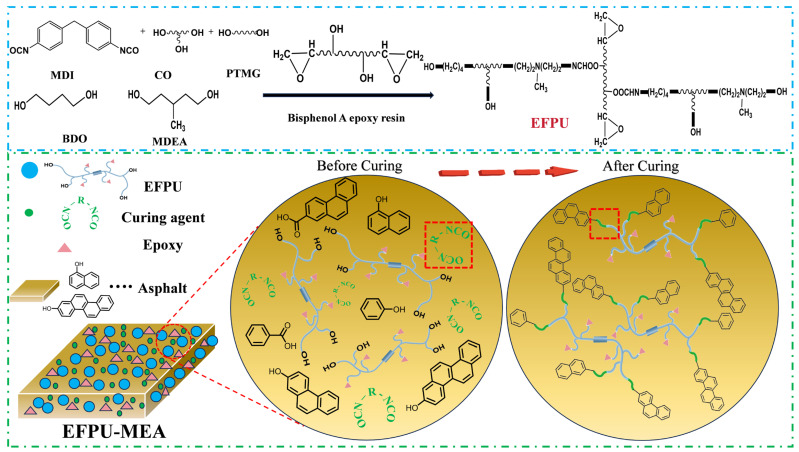
Schematic illustration of the modification mechanism for EFPU-modified emulsified asphalt (EFPU-MEA). (1. The ellipsis in the figure is due to the numerous classifications of active components in asphalt, and only representative structures are shown in the figure. 2. The red dashed box represents the mechanism of isocyanate curing agent as a “chemical bridge”).

To fundamentally address the limitations of conventional emulsified asphalt, such as an insufficient comprehensive performance and weak interfacial adhesion, this study proposes a “Physical–Chemical” synergistic reinforcement strategy. The molecular structure of the EFPU used and its modification principle for asphalt are illustrated in Figure 9.

In terms of molecular design, the EFPU is engineered to achieve an “intermolecular hybrid structure” by introducing epoxy groups into the polyurethane chains. Physically, the polyurethane segments provide flexibility, while the epoxy groups contribute rigidity and adhesion performance. This balanced structure endows the EFPU with essential toughness, mechanical strength, and adhesive potential, thereby significantly enhancing the comprehensive performance of the emulsified asphalt.

Simultaneously, regarding chemical modification, a curing agent is introduced to establish chemical bonding. Since the EFPU chains are completely hydroxyl-terminated, the externally added isocyanate curing agent acts as a chemical bridge. Specifically, the highly reactive isocyanate groups (-NCO) of the curing agent react vigorously with the terminal hydroxyl groups (-OH) of the EFPU chains, as well as with the inherent active hydrogen functional groups within the asphalt matrix. This reaction transforms the interface from a weak physical boundary into a robust chemical bond, thereby significantly increasing the surface free energy and the work of adhesion.

To comprehensively validate this proposed mechanism and its resulting performance, a systematic evaluation was conducted. Basic property tests and quantitative pull-out/shear experiments were employed to verify the macroscopic engineering performance and interfacial bond strength. Subsequently, the underlying micro-mechanisms were rigorously elucidated. Finally, the road performance is verified to ensure its engineering applicability.

### 3.1. Basic Performance of EFPU-MEA

EFPU-modified emulsified asphalt (EFPU-MEA) was prepared by incorporating EFPU into the base emulsion. To comprehensively assess its engineering applicability, a systematic evaluation was conducted covering mechanical properties, thermal stability (heat resistance and low-temperature flexibility), corrosion resistance, and conventional interfacial adhesion via the boiling water method.

#### 3.1.1. Mechanical Properties

**Figure 10:**
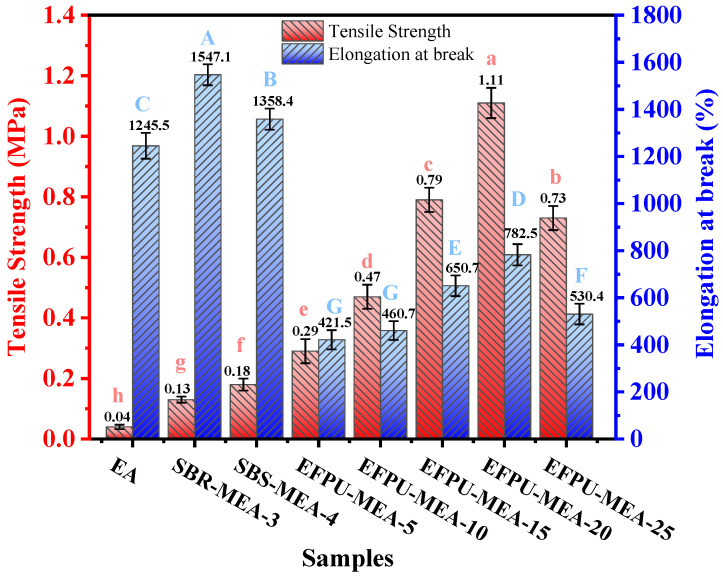
Tensile strength and elongation at break of different modified asphalt samples.

The mechanical property test results for the different samples are presented in Figure 10. The incorporation of EFPU significantly enhances the mechanical strength of the asphalt material. With increasing EFPU content, the tensile strength of the modified asphalt exhibited an initial upward trend followed by a decline, reaching a remarkable peak of 1.11 MPa at a 20% dosage. The tensile strength values at all EFPU dosage levels significantly outperformed those of conventional SBS-MEA (0.18 MPa) and SBR-MEA (0.13 MPa). Regarding the elongation at break, the EFPU-MEA series showed a similar trend, peaking at 782.5% with 20% EFPU. Although these elongation values remain lower than those of the base EA (1245.5%), SBR-MEA (1547.1%), and SBS-MEA (1358.4%), the EFPU-MEA preserves a sufficient deformation capability while delivering vastly superior structural strength. However, when the EFPU content further increased to 25%, both the tensile strength and elongation at break dropped sharply to 0.73 MPa and 530.4%, respectively. This simultaneous decline is attributed to severe particle agglomeration, as directly evidenced by the fluorescence microscopy (FM) observations in Section 3.3.1. Therefore, an EFPU content of 20% is identified as the optimal dosage to achieve the best balance between rigidity and flexibility.

#### 3.1.2. Heat Resistance

**Figure 11:**
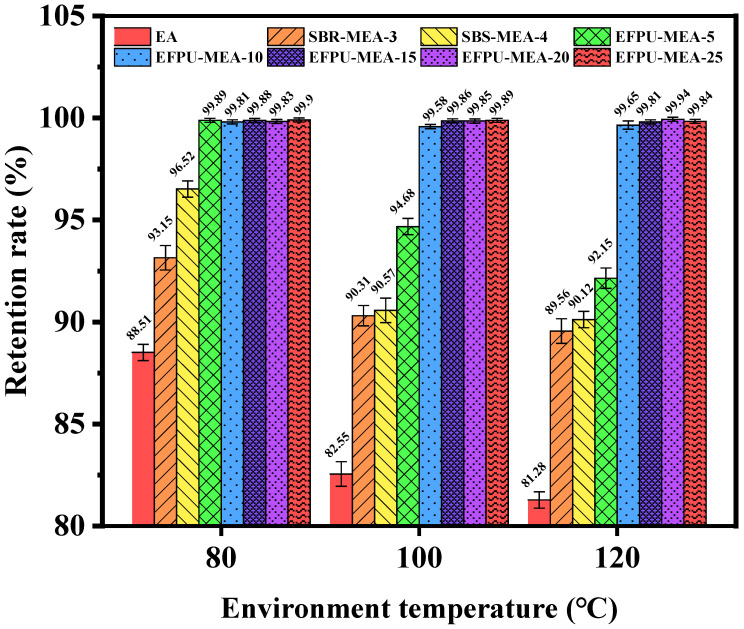
Quality retention rates of different samples at various temperatures.

**Figure 12:**
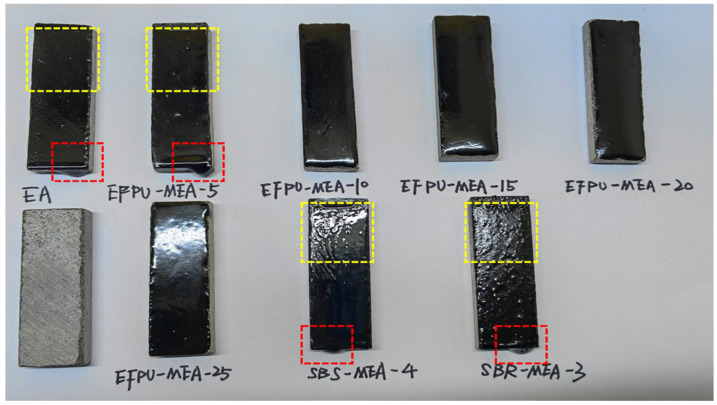
Condition of different samples at 120 °C. (The red dashed box shows the droplet marks of the sample, and the yellow dashed box shows the slip marks of the sample.)

Figure 11 and Figure 12 illustrate the heat resistance of the modified asphalt samples across various modifier dosages. The macroscopic high-temperature stability of the binder improved progressively as the EFPU content increased. Within the 80–120 °C range, the base EA, SBR-MEA-3, and SBS-MEA-4 and the low-dosage EFPU-MEA-5 exhibited pronounced softening and viscous flow, corresponding to a sharp decline in their mass retention rates. Although prolonged exposure to elevated temperatures naturally induces thermo-oxidative aging (which typically stiffens the binder), the severe dripping observed in these control groups confirms that physical thermal softening and the structural collapse of the viscoelastic matrix were the primary modes of coating failure. Visually, this failure manifested as localized matrix flowing and interfacial slipping, as marked by the red and yellow boxes in Figure 12. Conversely, samples containing 10% or more EFPU demonstrated zero macroscopic flow even at elevated temperatures, maintaining a near-perfect mass retention rate of approximately 99.9%. This indicates that crossing the 10% EFPU threshold establishes a sufficiently robust internal crosslinked network, which effectively resists thermal creep and maintains strong interfacial adhesion, far outperforming conventional SBS and SBR modification systems.

#### 3.1.3. Low-Temperature Flexibility

**Figure 13:**
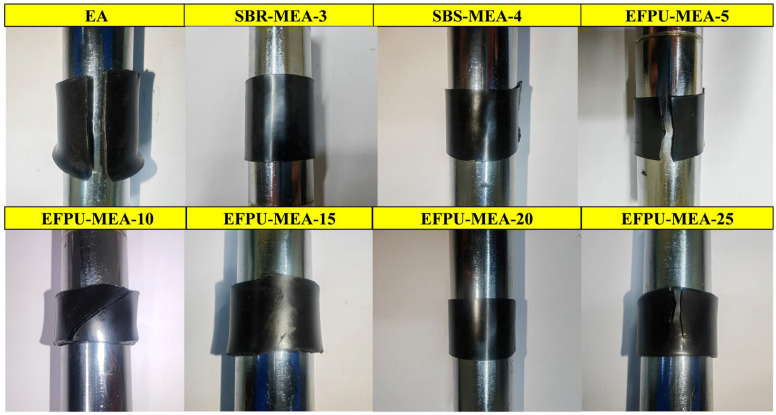
Photographs of modified emulsified asphalt samples after low-temperature (−15 °C) flexibility testing.

The low-temperature flexibility test results at −10 °C are presented in Figure 13. After bending at a low temperature, no cracking was observed on the surfaces of the SBS-MEA-4, SBR-MEA-3, EFPU-MEA-15, and EFPU-MEA-20 specimens, indicating that they possess good low-temperature crack resistance. In contrast, the EA and low-dosage EFPU-MEA-5 specimens underwent brittle fracturing, while visible cracks appeared on the surfaces of the EFPU-MEA-10 and EFPU-MEA-25 specimens. These results suggest that an optimal EFPU dosage range of 15–20% exists for achieving the best low-temperature performance in the modified asphalt. When the dosage is too low (≤5%), the modifier content is insufficient to effectively suppress the low-temperature brittleness of the asphalt. On the other hand, an excessively high dosage (e.g., 25%) leads to reduced toughness, which is attributed to the severe agglomeration of EFPU. As demonstrated by the fluorescence microscopy (FM) analysis in Section 3.3.1, the formation of large polymer clusters at this high dosage disrupts the structural homogeneity of the asphalt matrix. These clusters act as stress concentration sites that facilitate crack initiation and propagation at low temperatures, ultimately deteriorating the material’s flexibility.

#### 3.1.4. Corrosion Resistance

**Figure 14:**
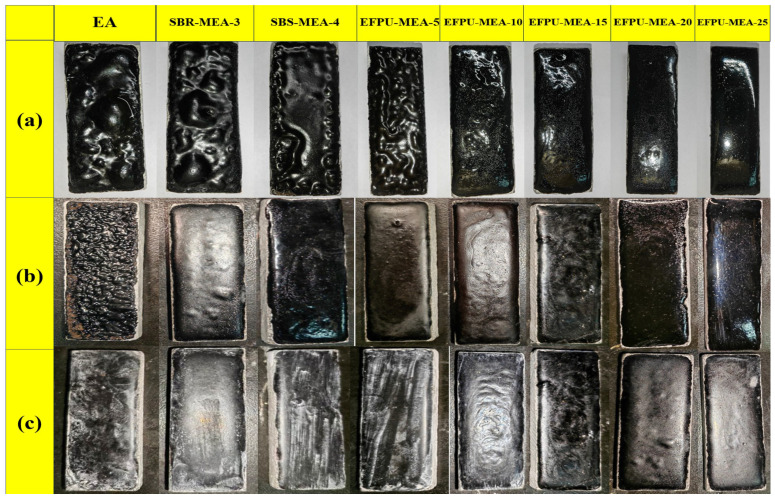
Photographs of various samples after (**a**) acid corrosion, (**b**) alkali corrosion, and (**c**) salt corrosion resistance test.

The durability of various samples in acidic, alkaline, and saline media was evaluated, with results shown in Figure 14. In acidic conditions, EA, SBS-MEA-4, SBR-MEA-3, and EFPU-MEA-5 exhibited blistering and film stripping due to the H+-induced dissolution of calcium carbonate in the aggregate, which weakened the interface. In contrast, samples with >10% EFPU content remained intact, as the EFPU-curing agent–asphalt network effectively blocked H^+^ penetration. In alkaline exposure, EA showed surface failure, whereas all polymer-modified asphalt samples remained intact due to the alkali resistance of polymer chains. Under salt erosion, EA, SBS-MEA-4, SBR-MEA-3, and EFPU-MEA-5 displayed crystalline deposits, indicating an insufficient resistance to NaCl crystallization stress [37]. However, samples with ≥10% EFPU maintained clean surfaces, effectively preventing salt accumulation and infiltration. These results confirm that when the EFPU content is ≥10%, the EFPU-MEA system can form a stable crosslinked network structure, maintaining superior interfacial integrity and durability in various corrosive environments, with a performance significantly outperforming conventional polymer-modified systems.

#### 3.1.5. Traditional Adhesive Performance—Boiling Water Method

**Figure 15:**
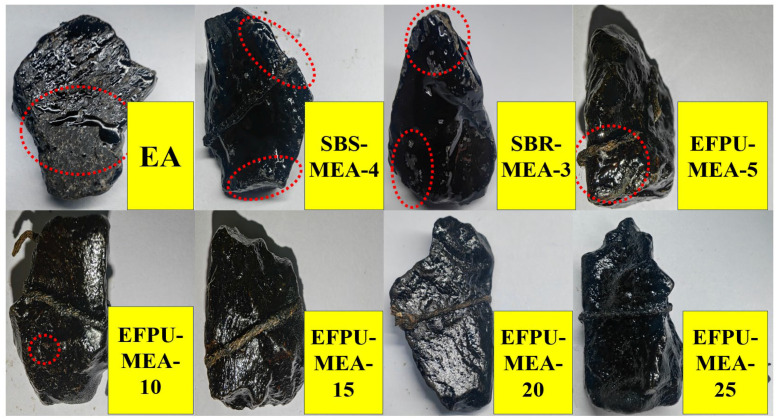
Boiling water test results for adhesion performance. (The red dashed box indicates the location where the sample fell off).

The boiling water test results (Figure 15) reveal significant differences in the adhesion performance at the asphalt–aggregate interface among the samples. The EA sample exhibited severe stripping under boiling water due to its reliance on physical adsorption for interfacial bonding. While SBS- and SBR-modified systems showed improved resistance to stripping owing to physical toughening effects, localized interfacial failure still occurred. At a 5% EFPU content, stripping was only observed at stress-concentrated edges of the aggregate, indicating a markedly enhanced interfacial adhesion. When the EFPU content reached 10% or higher, the asphalt film remained largely intact with no significant detachment. These results demonstrate that the EFPU-MEA system offers superior adhesion performance and moisture resistance under boiling conditions compared to conventional systems.

### 3.2. Interfacial Bond Performance of the “Sandwich” Structure

#### 3.2.1. Effect of Spraying Quantity

**Figure 16:**
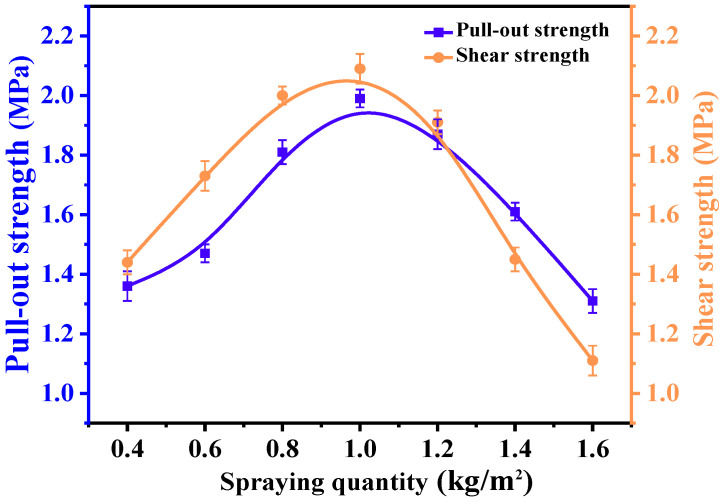
Effect of spraying quantity on pull-out and shear strength of modified emulsified asphalt.

**Figure 17:**
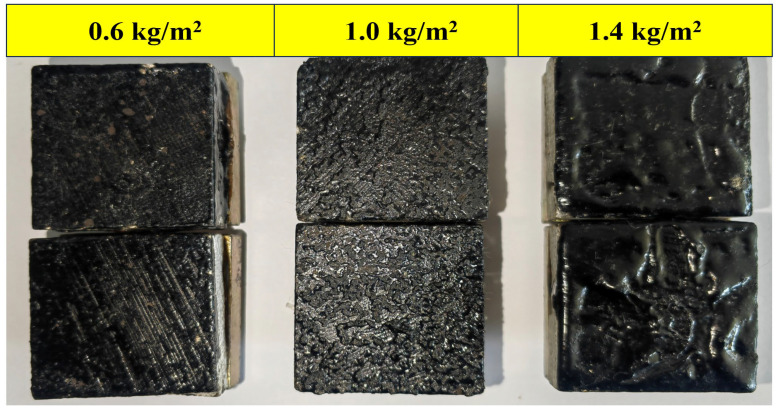
Fracture surface images of pull-out test specimens with different spraying quantities.

As shown in Figure 16, both pull-out and shear strengths exhibited a distinct trend of initially increasing and then decreasing with the spraying quantity. The empirical data clearly demonstrates that the optimal performance for both macroscopic indicators is concentrated near 1.0 kg/m^2^. Interestingly, the visual trend indicates that the pull-out strength tends to reach its peak at a slightly higher spraying quantity compared to the shear strength. This variation originates from the fundamental differences between the two loading mechanisms: the pull-out strength benefits from a slightly thicker and more flexible asphalt film to facilitate stress dissipation, whereas the shear strength requires a relatively thinner and stiffer film for effective load transfer [38,39]. Furthermore, the morphology of the failure surfaces under different spraying quantities (Figure 17) revealed that excessive asphalt leads to the formation of an interfacial sliding layer, while insufficient asphalt fails to achieve complete aggregate coverage and form a continuous film. Both extreme conditions result in a compromised bond performance. Based on these empirical findings and the practical engineering need to balance interfacial toughness with shear load transfer efficiency, the spraying quantity was directly optimized at 1.0 kg/m^2^ for subsequent experiments. This specific value effectively captures the performance peak zone, thereby establishing a reliable experimental basis for the systematic investigation of the pull-out and shear performance of EFPU-MEA under various influencing factors.

#### 3.2.2. Effect of EFPU Content

**Figure 18:**
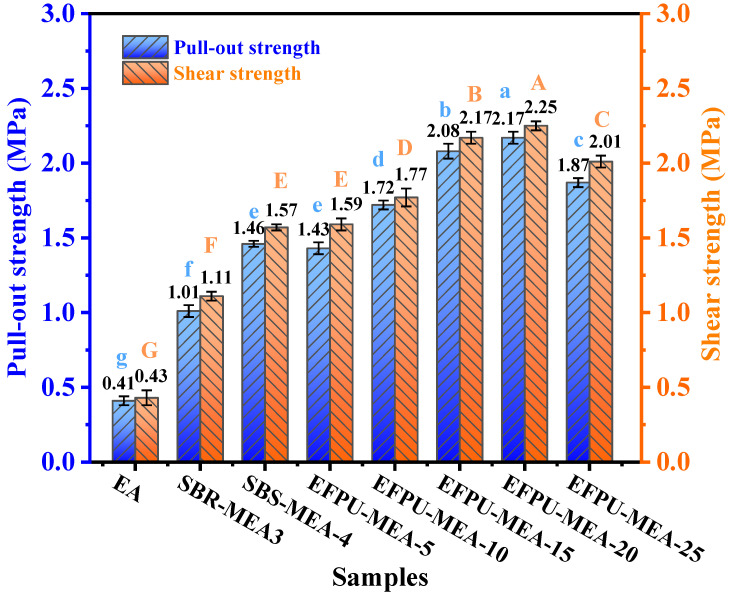
Effect of EFPU content on the pull-out strength and shear strength of modified emulsified asphalt.

As shown in Figure 18, compared to unmodified emulsified asphalt (EA), all polymer-modified systems (SBR, SBS, and EFPU) demonstrated significant improvements in both the pull-out strength and shear strength, indicating that polymer modification effectively enhances the bond performance at the asphalt–aggregate interface. Further analysis revealed that, as the EFPU content increased from 5% to 20%, the pull-out and shear strengths of EFPU-MEA showed continuous growth, with the most pronounced enhancement observed within the 15–20% dosage range. The maximum values (pull-out: 2.17 MPa; shear: 2.25 MPa) were achieved at a 20% EFPU content. However, a slight decrease in both the pull-out strength and shear strength occurred when the dosage was further increased to 25%, which may be attributed to the potential local agglomeration of EFPU within the asphalt matrix. Based on the observed strength variation patterns, EFPU dosage levels of 15% and 20%, which offer representative and outstanding comprehensive performances, were selected for subsequent experiments.

#### 3.2.3. Effect of Temperature

**Figure 19:**
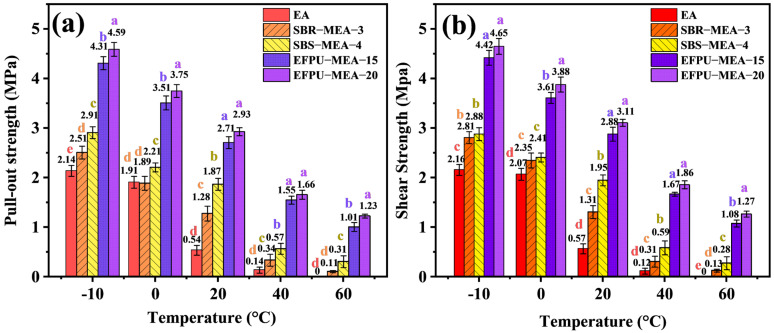
Effect of temperature on the pull-out and shear strengths of the modified emulsified asphalt: (**a**) pull-out strength; (**b**) shear strength.

Figure 19 presents the test results of pull-out strength and shear strength for different MEA samples over a temperature range of −10 °C to 60 °C. The results demonstrate that the EFPU-MEA system exhibits the most superior bonding performance across this broad temperature spectrum. At the high temperature of 60 °C, the pull-out strengths of EFPU-MEA-15 and EFPU-MEA-20 were 1.01 MPa and 1.23 MPa, respectively, and the shear strengths were 1.08 MPa and 1.27 MPa. These values are significantly higher than those of SBS-MEA (pull-out strength: 0.31 MPa; shear strength: 0.28 MPa) and SBR-MEA (pull-out strength: 0.11 MPa; shear strength: 0.13 MPa), while the base asphalt lost its bonding capacity at this temperature. Under the low-temperature condition of −10 °C, EFPU-MEA maintained high strength levels, with pull-out strengths ranging from 4.31 MPa to 4.59 MPa and shear strengths between 4.42 MPa and 4.65 MPa, markedly outperforming conventional SBR- and SBS-modified emulsified asphalt systems. These findings indicate that EFPU-MEA possesses an excellent interfacial bonding performance and thermal stability over a wide temperature range.

#### 3.2.4. Effect of Water Immersion

**Figure 20:**
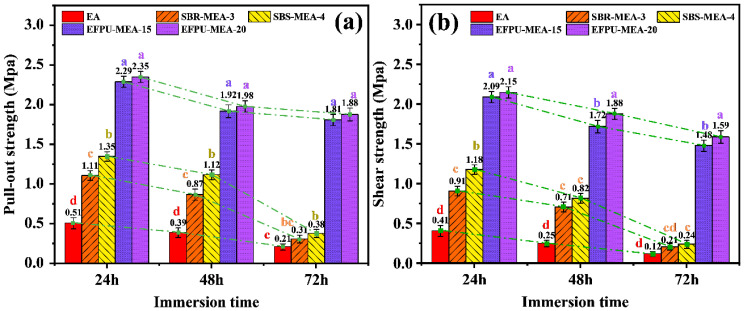
Effect of water immersion on the pull-out and shear strengths of the modified emulsified asphalt: (**a**) pull-out strength; and (**b**) shear strength. The green dashed lines indicate the variation trends of the strengths over the immersion time.

**Figure 21:**
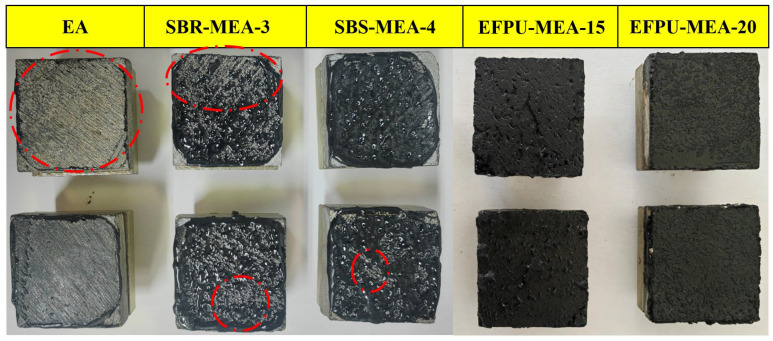
Photographs of the failure modes of the modified emulsified asphalt after water immersion. (The red dashed lines indicate the areas of exposed aggregate.)

Under water immersion conditions (Figure 20 and Figure 21), all modified emulsified asphalt systems showed a reduced bond strength with extended immersion time, but with significant differences in moisture resistance. While conventional systems (SBS-MEA-4 and SBR-MEA-3) exhibited severe strength reductions of 71.9–79.7%, the EFPU-modified system demonstrated superior water stability. EFPU-MEA-20 maintained much higher strength retention, with reductions of only 20.0% in pull-out strength and 26.0% in shear strength after 72 h immersion. Critical immersion period analysis (48–72 h) revealed accelerated deterioration in conventional systems (64.4–66.1% reduction) compared to merely 5.1% for EFPU-MEA-20. Failure mode analysis (Figure 21) further explained these differences: EA showed adhesive failure, SBR/SBS exhibited mixed failure, while EFPU maintained cohesive failure, indicating that its interfacial bond strength exceeds the cohesive strength of asphalt itself. These results confirm that EFPU modification significantly enhances the moisture damage resistance of emulsified asphalt.

#### 3.2.5. Effect of Freeze–Thaw Cycle

**Figure 22:**
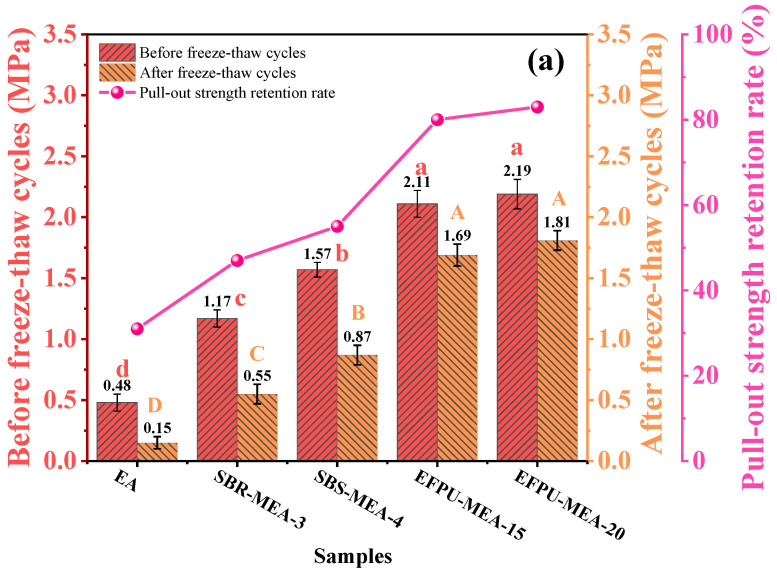

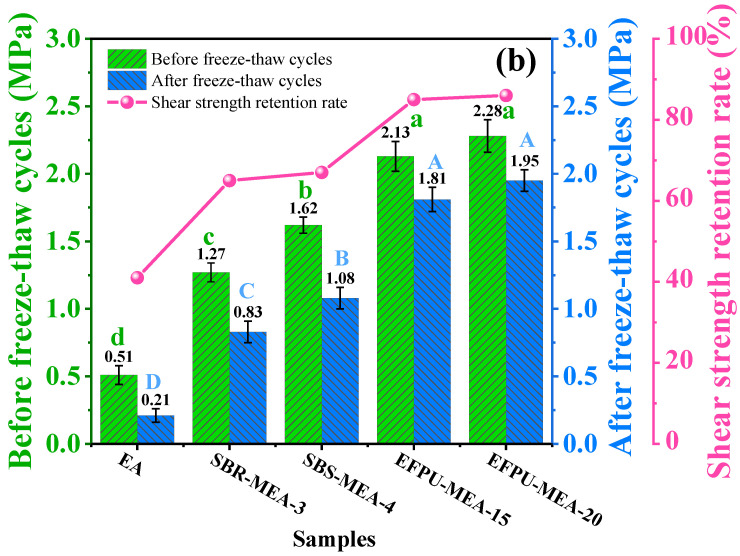
Pull-out and shear strength retention of various modified emulsified asphalts after freeze–thaw cycles: (**a**) pull-out strength; and (**b**) shear strength.

**Figure 23:**
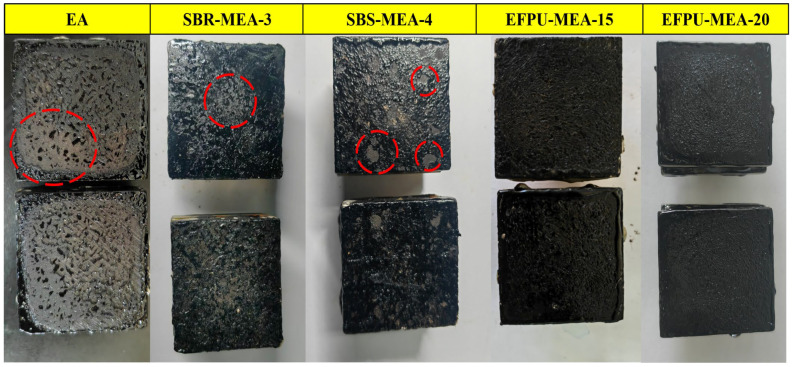
Photographs of the failure modes of various modified emulsified asphalts after freeze–thaw cycles. The red dashed lines indicate the internal voids or areas of exposed aggregate.

The freeze–thaw cycle test results (Figure 22 and Figure 23) demonstrated the superior resistance of the EFPU-modified system, which maintained residual strength rates of 83% (pull-out strength) and 86% (shear strength)—significantly higher than conventional modified systems (47–67%) and the base asphalt (31–41%). In terms of absolute strength, EFPU-MEA-20 retained strengths of 1.81 MPa (pull-out strength) and 1.95 MPa (shear strength), values 9.3–12.1 times greater than the EA system and 2.1–3.3 times those of conventional systems. Fracture surface analysis revealed cohesive failure in EFPU specimens, confirming enhanced interfacial bonding that effectively resists moisture and freeze–thaw damage. These results affirm the exceptional freeze–thaw durability of EFPU-MEA.

### 3.3. Modification Mechanisms of EFPU-MEA

#### 3.3.1. Morphological Analysis via Fluorescent Microscope (FM)

**Figure 24:**
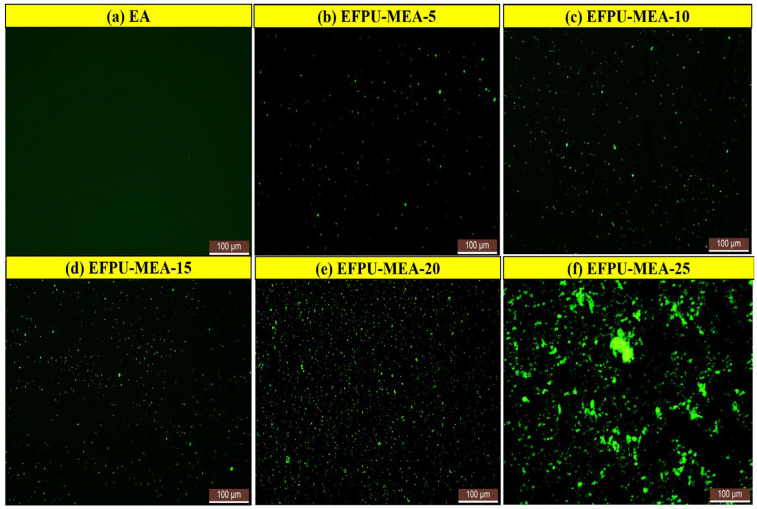
FM images of emulsified asphalts with different EFPU contents.

FM observations (Figure 24) revealed the dispersion state of EFPU within the asphalt matrix. As shown in Figure 24a, the unmodified EA sample exhibited no significant fluorescence signal. At low-dosage levels (EFPU-MEA-5, Figure 24b; EFPU-MEA-10, Figure 24c), weak but uniformly distributed green fluorescence was observed, showing the discrete punctate distribution of EFPU. When the dosage increased to 15% and 20% (Figure 24d,e), both the dispersion density and distribution homogeneity of EFPU significantly improved. Notably, at 25% dosage (Figure 24f), localized fluorescence enrichment indicated the partial agglomeration of EFPU. These microstructural characteristics align well with the macroscopic performance results: the uniformly dispersed phase structure in EFPU-MEA-15 and EFPU-MEA-20 correlates with their superior performance, while the agglomeration in EFPU-MEA-25 explains its performance degradation trend. The results demonstrate that the optimal dosage range for EFPU in asphalt is 15–20%.

#### 3.3.2. Surface Energy Analysis

**Figure 25:**
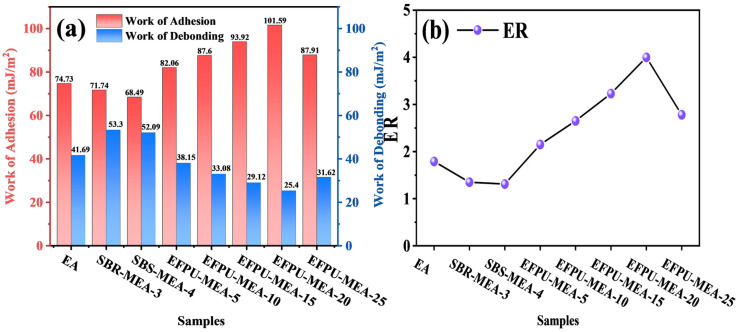
(**a**) Calculation results of adhesion work and debonding work. (**b**) Energy ratio results for different modified emulsified asphalts.

Based on the comprehensive analysis of adhesion work, debonding work, and the energy ratio, EFPU modification demonstrates remarkable effectiveness in enhancing the interfacial performance of emulsified asphalt. As shown in Figure 25a, with the increase in EFPU content from 0% to 20%, the adhesion work exhibits a significant and continuous growth from 74.73 to 101.59 mJ/m^2^, while the debonding work decreases substantially from 41.69 to 25.40 mJ/m^2^. Thermodynamically, debonding work in this context represents the energy released (i.e., the driving force) when water displaces the asphalt film from the aggregate surface. Therefore, the drastic reduction in debonding work signifies a much weaker thermodynamic tendency for moisture-induced stripping.

This mechanistic trend indicates that EFPU modification not only strengthens the interfacial bond strength in dry conditions but also fundamentally improves the moisture resistance of the system. The energy ratio (ER) analysis (Figure 25b) further confirms this conclusion, showing a remarkable increase from 1.79 (EA) to 4.00 (EFPU-MEA-20), representing an approximately 123% enhancement. According to established thermodynamic evaluations, mixtures with an ER higher than 1.5 are considered to possess superior moisture resistance. Notably, while the base emulsified asphalt exhibits an adequate baseline moisture resistance (ER = 1.79), the interfacial performance of conventional modified systems such as SBS-MEA (ER = 1.31) and SBR-MEA (ER = 1.35) deteriorates, falling below the 1.5 threshold. They exhibit even lower ER values than the base asphalt due to the “polarity dilution” effect of non-polar rubber chains.

Surface energy parameters (Table 6) provide deep mechanistic insights into this enhanced performance. The incorporation of EFPU fundamentally alters the surface characteristics of asphalt, leading to a synergistic increase in both dispersive (γ_a_^d^) and polar (γ_a_^p^) components. For instance, EFPU-MEA-20 exhibits a γ_a_^d^ of 41.87 mJ/m^2^ and a γ_a_^p^ of 20.12 mJ/m^2^, which are substantial improvements compared to the base asphalt (γ_a_^d^: 28.65 mJ/m^2^; γ_a_^p^: 4.82 mJ/m^2^). The surge in γ_a_^p^ originates from the abundant polar functional groups (urethane and urea linkages) in EFPU molecules. Simultaneously, the improved γ_a_^d^ results from the formation of a dense polymer network at optimal dosages, which enhances the molecular cohesion and transient dipole interactions.

However, thermodynamic potential must be matched by physical stability. Corroborating FM observations (Figure 24) reveal that, while the 15–20% dosage achieves an optimal and uniform dispersion, an excessive dosage (25%) leads to significant polymer aggregation. This physical defect causes the surface energy parameters of EFPU-MEA-25 to recede (γ_a_^d^: 33.42 mJ/m^2^; γ_a_^p^: 12.55 mJ/m^2^), consequently leading to a decline in ER to 2.78. This confirms that the superior interfacial performance at a 20% dosage stems from a synergistic mechanism of chemical polarity enhancement and physical reinforcement via a well-distributed EFPU phase.

#### 3.3.3. Fourier Transform Infrared (FTIR) Spectrum Analysis

**Figure 26:**
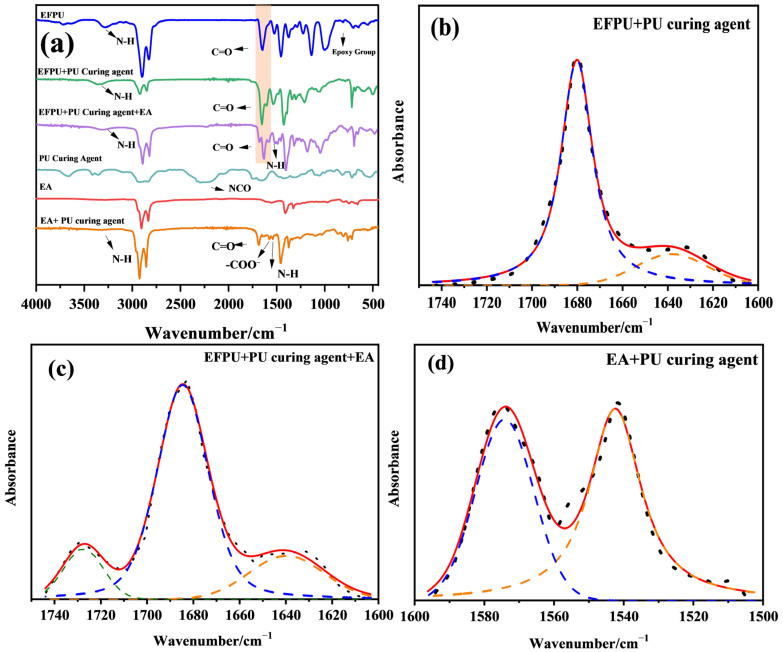
FTIR spectra of the modifiers and various emulsified asphalt samples: (**a**) modifiers and various emulsified asphalt samples; (**b**) curve-fitting deconvolution of the EFPU + PU curing agent system; (**c**) curve-fitting deconvolution of the EFPU + PU curing agent + EA ternary system; and (**d**) curve-fitting deconvolution of the EA + PU curing agent system. (1. The orange shaded areas highlight the peak variations among different samples. 2. The black dashed line represents the original spectrum, and the red line is the cumulative fitted curve. The orange, blue, and green dashed lines represent the individual deconvoluted sub-peaks).

To clarify the “chemical bridge” mechanism, FTIR analysis was performed via a systematic pairwise comparison, as shown in Figure 26 and Table 7. First, the interaction between the curing agent and EFPU was investigated. Pure EFPU exhibits a solitary peak at 1730 cm^−1^, corresponding to the C=O of urethane linkages. Upon the addition of the curing agent, this peak evolves into a distinct doublet. To quantitatively validate this, curve-fitting deconvolution (R^2^ = 0.9897) was applied, revealing that the doublet consists of peaks at 1679 cm^−1^ (relative area: 81.49%) and 1638 cm^−1^ (18.50%), assigned to strongly hydrogen-bonded urethane and newly formed urea linkages, respectively. This confirms that the -NCO groups of the curing agent react directly with the terminal hydroxyl groups of the EFPU chains to form a crosslinked network.

Second, the reaction between the curing agent and the base emulsified asphalt (EA) was analyzed. The spectrum of the pure curing agent shows a characteristic -NCO peak at 2270 cm^−1^, which completely disappears in the EA/curing agent blend, indicating full consumption of the isocyanate groups. Simultaneously, new peaks emerge at 3340 cm^−1^ (N–H stretching) and 1686 cm^−1^ (amide I). Given the spectral overlap with the inherent aromatic ring vibrations of the asphalt matrix (1550–1650 cm^−1^), the 1650–1500 cm^−1^ region was mathematically deconvoluted (R^2^ = 0.9639). The analysis successfully isolated the carboxylate ion absorption at 1574 cm^−1^ (relative area: 44.90%), generated by the reaction between -NCO and the asphalt’s carboxylic acid functionalities from the amide II band at 1542 cm^−1^ (55.09%). These observations provide robust quantitative evidence that the curing agent chemically bonds with the active components of the EA, although the extreme complexity of asphalt components necessitates future model compound studies for definitive verification.

Finally, the chemical modification mechanism in the EFPU-MEA ternary system was deeply analyzed. Following the introduction of EA, the C=O stretching region (1800–1600 cm^−1^) further differentiates into a triplet structure. Gaussian curve fitting (R^2^ = 0.9921) effectively resolved this envelope into three distinct subpeaks: 1727 cm^−1^ (relative area: 11.50%), 1685 cm^−1^ (69.89%), and 1640 cm^−1^ (18.59%). Mechanistically, the peak at 1727 cm^−1^ is ascribed to urethane C=O in a weak hydrogen-bonding environment, likely due to the plasticization effect of light asphalt components which disrupts polymer interchain interactions. Conversely, peaks at 1685 cm^−1^ and 1640 cm^−1^ correspond to strongly hydrogen-bonded urethane and urea domains. The emergence of these characteristic peaks, combined with the quantitatively validated pairwise results, provides compelling evidence for the formation of a complex, chemically crosslinked network bridging the EFPU and asphalt matrix.

### 3.4. Performance Tests for Asphalt Mixture

#### 3.4.1. Marshall Stability Test

**Figure 27:**
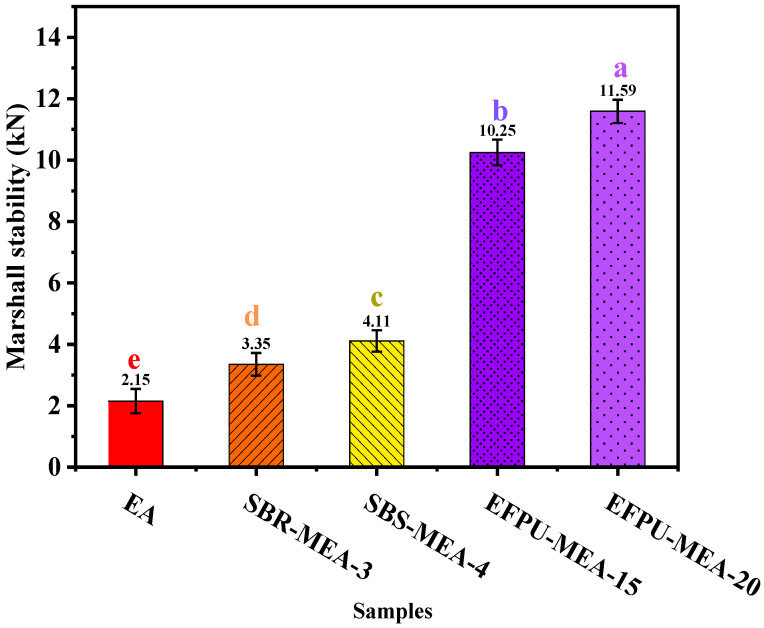
Marshall stability of asphalt mixtures with different modifiers.

The Marshall stability test results (Figure 27) demonstrate that EFPU significantly enhances the high-temperature stability of asphalt mixtures. The EFPU-MEA system exhibited markedly higher stability values compared to reference samples. Specifically, EFPU-MEA-15 and EFPU-MEA-20 achieved stability values of 10.25 kN and 11.59 kN, respectively, representing substantial improvements over both the unmodified base asphalt (EA: 2.15 kN) and conventional polymer-modified systems (SBR-MEA-3: 3.35 kN; SBS-MEA-4: 4.11 kN). These results validate the excellent high-temperature stability and strength of the EFPU-MEA system in pavement performance.

#### 3.4.2. Wet Track Abrasion Test

**Figure 28:**
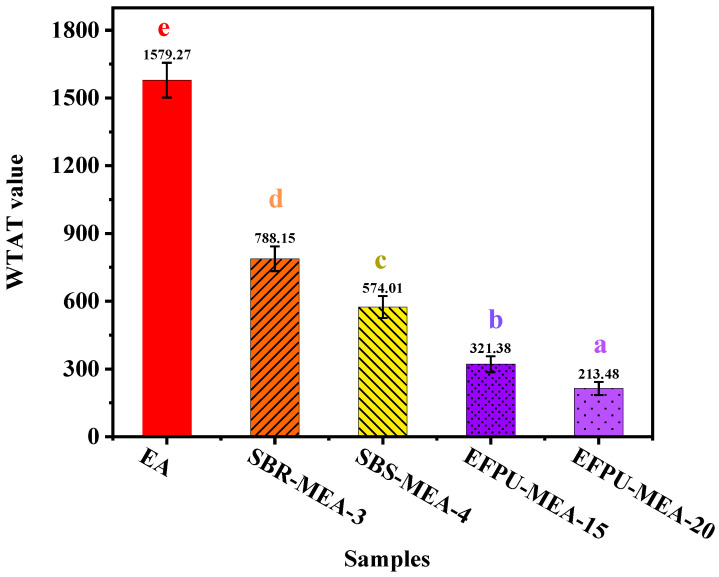
WTAT value of various samples after 6 days of wet wheel abrasion testing.

**Figure 29:**
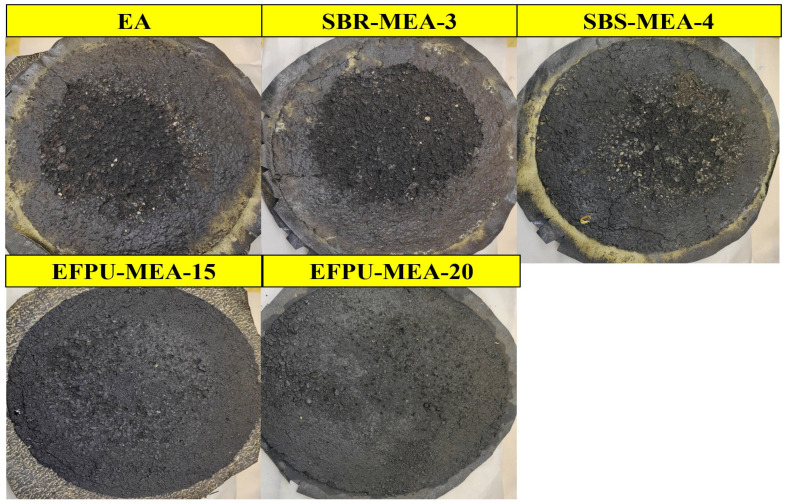
Visual appearance of samples after wet wheel abrasion testing.

The six-day wet track abrasion test results (Figure 28 and Figure 29) reveal significant differences in the wear resistance among the various modified emulsified asphalt systems. The unmodified base asphalt (EA) exhibited the highest abrasion value of 1579.28, while the EFPU-modified systems demonstrated markedly superior wear resistance. Specifically, EFPU-MEA-15 and EFPU-MEA-20 achieved abrasion values of 321.38 and 213.49, respectively, representing reductions of 79.7% and 86.5% compared to EA. Although the conventional modified systems, SBR-MEA-3 (788.15) and SBS-MEA-4 (574.01), showed some improvement over EA, their abrasion values remained significantly higher than those of the EFPU-modified systems. Observations of the worn sample surfaces (Figure 29) further indicated that the EFPU-modified samples exhibited only localized, slight grain loss, whereas the EA and conventional modified systems showed extensive peeling. These results confirm that the EFPU-modified system, owing to its excellent interfacial bonding performance, effectively resists the combined damaging effects of water erosion and mechanical wear.

#### 3.4.3. Analysis of Anti-Rutting Performance

**Figure 30:**
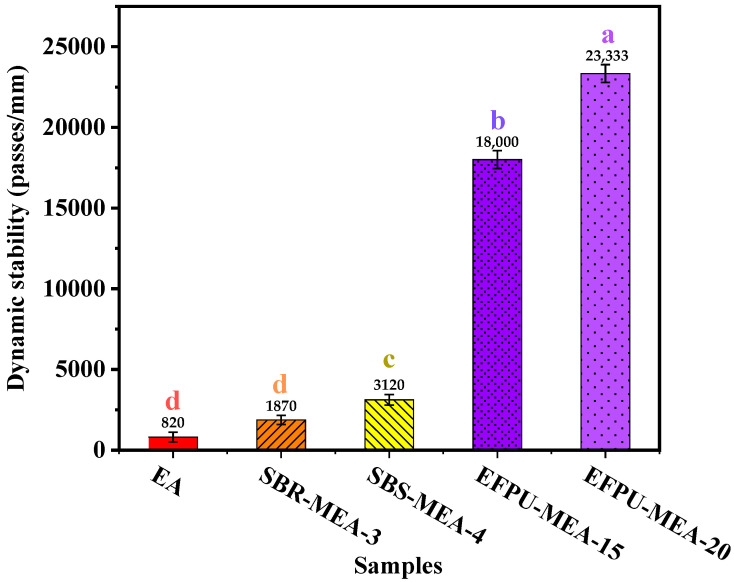
Dynamic stability of asphalt mixtures with different modified asphalt emulsions.

The dynamic stability test results for different samples are shown in Figure 30. It can be observed that the EFPU-modified emulsified asphalt (EFPU-MEA) exhibits significantly improved high-temperature rutting resistance compared to conventional systems. Specifically, the dynamic stability of EFPU-MEA-20 reaches 23,333 passes/mm, which is 7.5 times that of SBS-modified emulsified asphalt (3120 passes/mm); the dynamic stability of EFPU-MEA-15 also reaches 18,000 passes/mm, equivalent to 5.8 times that of the SBS system, demonstrating clear superiority over conventional modified systems and confirming its excellent high-temperature rutting resistance. Under high-temperature conditions, after the pavement load is removed, the EFPU in the system promotes timely elastic recovery of the pavement, reducing the accumulation of plastic deformation. Although high temperatures cause softening of the asphalt matrix, the strong interfacial bonding between EFPU and aggregate effectively inhibits the relative sliding and rearrangement of aggregate particles, thereby maintaining the stability of the aggregate skeleton and preventing skeleton instability and rut formation caused by the lubricating effect of asphalt.

## 4. Conclusions and Perspectives

In this study, an epoxy-functionalized waterborne polyurethane-modified emulsified asphalt (EFPU-MEA) was prepared. The macroscopic properties and interfacial adhesion mechanisms of the modified binder were systematically evaluated. The main conclusions are summarized as follows:

(1) The incorporation of EFPU significantly enhanced the basic performance of the emulsified asphalt. At the optimal dosage, EFPU-MEA achieved a tensile strength of 1.11 ± 0.05 MPa and an elongation at break of 782.5 ± 45%. Furthermore, it exhibited superior heat resistance, low-temperature flexibility, chemical corrosion resistance, and conventional boiling water adhesion compared to the unmodified and traditional polymer-modified binders. However, these improvements were evaluated using a specific base asphalt under standard, unaged conditions. The long-term aging effects and performance across a wider temperature spectrum warrant further systematic investigation.
(2) A limestone “sandwich” structure was utilized to quantitatively evaluate the interlayer pull-out and shear performance. Based on the test results, the optimal spraying quantity and modifier content were determined to be 1.0 kg/m^2^ and 15–20%, respectively. Under these parameters, the asphalt–aggregate interface maintained exceptional bond strength across a wide temperature range (−10 °C to 60 °C) and following severe water immersion and freeze–thaw cycles.
(3) The performance improvements are attributed to a “chemical–physical” synergistic mechanism. Fluorescence microscopy (FM) indicated that EFPU (at a 15–20% dosage) is uniformly dispersed within the asphalt matrix. Concurrently, FTIR confirmed the formation of a chemically crosslinked network rich in urethane and urea linkages. This internal crosslinking fundamentally alters the surface thermodynamics, leading to a notable increase in the polar component of the surface free energy. Consequently, the work of adhesion is increased, while the work of debonding is reduced, explaining the enhanced interfacial anchoring.
(4) Macroscopic mixture tests, including Marshall stability, wet track abrasion, and rutting resistance evaluations, verified the engineering durability of EFPU-MEA. The results demonstrate its practical application potential in preventive maintenance treatments for high-grade highways, such as micro-surfacing and slurry seals.

Finally, while this study comprehensively establishes the laboratory performance and fundamental interfacial mechanisms of the EFPU-MEA system, it should be acknowledged that these evaluations were conducted under controlled conditions. Future studies will focus on the long-term environmental degradation behaviors of the modified emulsion—specifically encompassing systematic UV aging and long-term oxidative aging experiments. Regarding chemical durability, it is speculated that prolonged UV exposure may act as “molecular scissors,” gradually breaking the polymer chains (urethane and urea linkages) and reducing the network density. Similarly, long-term oxidative aging may lead to the hardening and embrittlement of the EFPU phase, potentially weakening the interfacial adhesion over time. Furthermore, the construction of full-scale field pilot sections will be undertaken. These ongoing efforts will further validate the long-term in situ durability and fatigue resistance of the EFPU-MEA system under actual traffic loading and complex weather conditions.

## Figures and Tables

**Table 1:** Physical properties and chemical composition of the base asphalt.

Properties Physical Properties	Unit	Value	Test Method (JTG E20-2011)
Penetration (25 °C)	0.1 mm	68	T 0604
Softening Point	°C	49	T 0606
Ductility (10 °C)	cm	39	T 0605
Saturates	%	7.2	T 0618
Aromatics	%	52.4	T 0618
Resins	%	20.6	T 0618
Asphaltenes	%	19.8	T 0618
Acid Value	mg KOH/g	0.764	T 0629

**Table 2:** Properties of emulsified asphalt.

Emulsified Asphalt Evaporation Residue	Unit	Test Results	Standard Requirement	Method (JTG E20-2011)
Solid content	%	58.5	>50	T 0651
Penetration (25, 100 g, 5 s)	0.1 mm	63	20–200	T 0606
Ductility (15 °C, 5 cm⋅min^−1^)	cm	71.1	≥40	T 0605
Storage stability	%	0.5	≤1 (1 day)	T 0655
		3.4	≤5 (5 days)	T 0655

**Table 3:** Properties of three polymer emulsions.

Technical Indicators	SBR Latex	SBS Latex	EFPU
Appearance	White	White	Transparent with a bluish tinge
Solid content (%)	40 ± 1	30 ± 2	35 ± 2
pH	3.7	3.5	4.4
Ionic type	Cationic	Cationic	Cationic
Viscosity (Pa·s)	31.1	320.5	22.5

**Table 4:** Properties of polyurethane curing agent.

Technical Indicators	Results
NCO (%)	21%
Appearance	Colorless transparent liquid
Solid content (%)	100
Viscosity (Brookfield, mPa·s)	≤3000

**Table 5:** Typical formulation and key synthesis parameters for the hydroxyl-terminated EFPU emulsion.

Raw Materials	Description	Mass (g)	Moles (mol)	Function
MDI-50	Liquefied MDI	31.7	0.1268	Hard segment
PTMG	Mn = 1000	23.95	0.024	Soft segment
Castor Oil	USP Grade	5.56	0.006	Crosslinker
MDEA	-	11.12	0.0935	Cationic center
BDO	-	0.71	0.0079	Chain extender
E-20	Bisphenol-A Epoxy	6.12	-	Modifier
Acetic Acid		6.41	0.1122	Neutralizer
Overall R value	NCO/OH	0.95	-	Stoichiometric ratio
Epoxy Dosage	m(Epoxy)/m(PU)	7.00%	-	Modification degree

**Table 6:** Contact angles and surface free energy parameters of the modified asphalt binders.

Samples	Contact Angle (Water)	Contact Angle (Formamide)	Contact Angle (Glycerol)	γ_a_^d^ (mJ/m^2^)	γ_a_^p^ (mJ/m^2^)	γ_a_ (mJ/m^2^)
EA	83.3°	61.2°	69.5°	28.65	4.82	33.47
SBR-MEA-3	94.8	67.6°	78.0°	33.5	0.82	34.3
SBS-MEA-4	96.4°	71.3°	80.8°	37.94	0.91	30.9
EFPU-MEA-5	77.7°	53.8°	63.2°	32.61	7.41	40.02
EFPU-MEA-10	68.3°	43.2°	52.7°	34.13	11.42	45.55
EFPU-MEA-15	60.4°	34.2°	43.6°	37.18	15.46	52.64
EFPU-MEA-20	52.1°	24.4°	32.2°	41.87	20.12	61.99
EFPU-MEA-25	67.0°	42.1°	51.4°	33.42	12.55	45.97

**Table 7:** FTIR deconvolution parameters and peak assignments for the different reaction systems.

	Peak Position	FWHM	Relative Area	R^2^
EFPU + PU curing agent	1638.25 1679.21	37.09 15.24	18.50 81.49	0.9897
EFPU + PU curing agent + EA	1639.77 1684.60 1727.45	37.60 25.51 20.01	18.59 69.89 11.50	0.9921
EA + PU curing agent	1542.42 1574.17	16.37 19.03	55.09 44.90	0.9639

## Data Availability

Data will be made available on request.

## Supplementary Materials

The following supporting information can be downloaded at: https://www.mdpi.com/article/10.3390/polym18060719/s1, Figure S1: Effect of polyurethane curing agent dosage on the tensile strength and elongation at break of pure EFPU films.
