# Supplementary material for: Enhancing the Comprehensive Performance and Interfacial Adhesion of Emulsified Asphalt Using an Epoxy-Functionalized Waterborne Polyurethane
Source: Polymers (Basel). 2026 Mar 16;18(6):719. doi: 10.3390/polym18060719 (PMC13030301; doi:10.3390/polym18060719)
Supplement: Supplementary file 1 [file polymers-18-00719-s001.zip › polymers-4194777-supplementary.pdf]

Supplementary Material

Enhancing the Comprehensive Performance and Interfacial Adhesion of Emulsified Asphalt Using an Epoxy-Functionalized Waterborne Polyurethan

Yifan Liu [a], Zhenhao Cao [a], Minghao Mu,[b] Zheng Wang,[b] Jia Wang [a],

Yanyan Zhang [a], Kunyu Wang [a], Yang Liu [c], Xue Li[a,d,*]

Optimization of Polyurethane Curing Agent Dosage

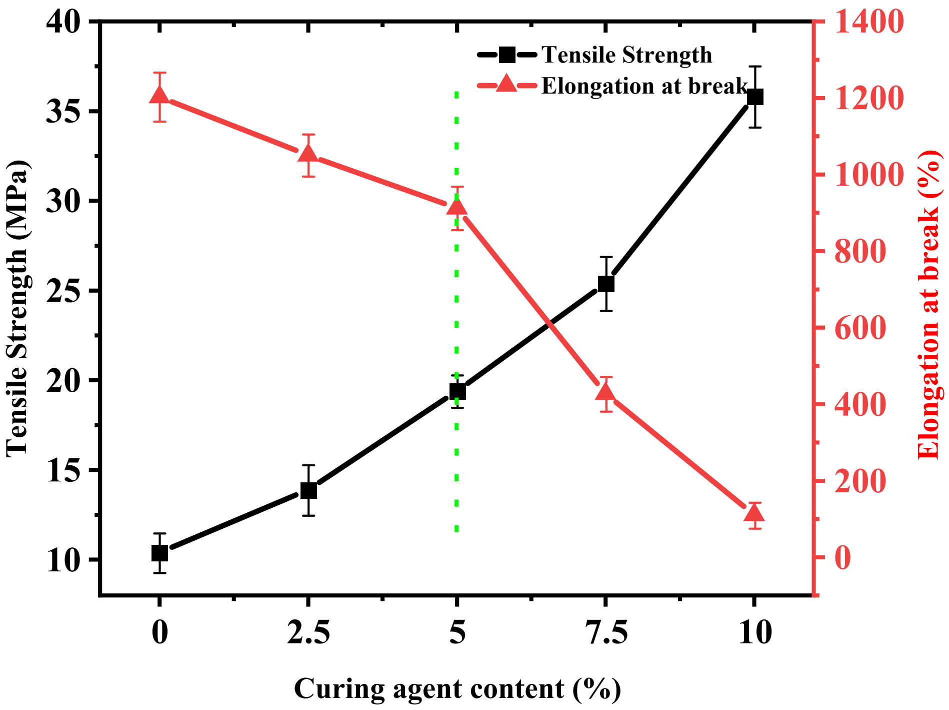

Figure S1. Effect of polyurethane curing agent dosage on the tensile strength and elongation at break of pure EFPU films.

To determine the optimal dosage of the polyurethane curing agent, a sensitivity analysis was conducted on pure EFPU films. The mechanical properties were evaluated with curing agent dosages ranging from 0% to 10% (by mass of EFPU). As shown in

Figure S1, the tensile strength exhibited a continuous upward trend with increasing dosage, confirming the effective enhancement of crosslinking density. However, a critical trade-off was observed in ductility: while the elongation at break remained excellent (>900%) at 5% dosage, it dropped sharply to 427.1% at 7.5% and further to 110.8% at 10%, indicating a transition to brittleness. Consequently, 5% was identified as the optimal dosage, effectively balancing mechanical strength and ductility to meet the requirements for pavement applications.
